# Dynamics of high viscosity contrast confluent microfluidic flows

**DOI:** 10.1038/s41598-017-06260-6

**Published:** 2017-07-19

**Authors:** Michael E. Kurdzinski, Berrak Gol, Aaron Co Hee, Peter Thurgood, Jiu Yang Zhu, Phred Petersen, Arnan Mitchell, Khashayar Khoshmanesh

**Affiliations:** 1 School of Engineering, RMIT University, Melbourne, Victoria Australia; 2 School of Media and Communication, RMIT University, Melbourne, Victoria Australia

## Abstract

The laminar nature of microfluidic flows is most elegantly demonstrated via the confluence of two fluids forming two stable parallel flows within a single channel meeting at a highly stable interface. However, maintenance of laminar conditions can become complicated when there is a large viscosity contrast between the neighbouring flows leading to unique instability patterns along their interface. Here, we study the dynamics of high viscosity contrast confluent flows – specifically a core flow made of highly viscous glycerol confined by sheath flows made of water within a microfluidic flow focusing system. Our experiments indicate the formation of tapered core structures along the middle of the channel. Increasing the sheath flow rate shortens the tapered core, and importantly induces local instability patterns along the interface of core-sheath flows. The dynamics of such tapered core structures is governed by the intensity of instability patterns and the length of the core, according to which the core structure can experience stable, disturbed, broken or oscillated regimes. We have studied the dynamics of tapered core structures under these regimes. In particular, we have analysed the amplitude and frequency of core displacements during the broken core and oscillating core regimes, which have not been investigated before.

## Introduction

The laminar nature of microfluidics is most elegantly demonstrated via the confluence of two fluids forming two stable parallel flows within a single channel meeting at an extremely stable interface^1^. If the two fluids are miscible then mixing will eventually occur due to diffusion; if the two fluids are immiscible (e.g. water/oil or air/water) then the parallel flows can be sustained as stable indefinitely so long as laminar conditions are maintained. The widths of these two parallel flows can be precisely determined according to the ratio of the velocity and viscosity of the two fluids. This ability to control the width of parallel flows has led to the technique of flow focusing where one flow is ‘sheathed’ by two surrounding flows of similar properties. This technique is particularly significant for flow cytometry^2, 3^, creating annular co-flow of liquids^4^ and lateral migration of suspended particles (e.g. colloidal particles and DNA molecules)^5^.

For miscible liquids, the conditions for laminar conditions depend on both the velocity and viscosity of the fluids. Maintenance of laminar conditions can become complicated when there is a large viscosity contrast between the neighbouring flows leading to unique instability patterns^6–9^, as comprehensively reviewed by Sahu *et al*.^10^. For example, both experimental^11–13^ and numerical^14^ analyses have shown the existence of pearl and mushroom instability patterns when applying two miscible liquids into a circular pipe with the viscous liquid acting as the sheath (annular) flow. Likewise, it has been shown both experimentally^15^ and numerically^16^ that applying two immiscible liquids into a circular pipe with the viscous liquid acting as the core flow is associated with bamboo instability patterns at the interface of the liquids. In particular, the stability of core-annular flows is important for transporting heavy and extra-heavy crude oil within a sheath of lubricating water^17^, which is used as an effective means for reducing friction losses through long pipelines^18–20^.

Over the last decade, Cubaud *et al*.^21, 22^ have comprehensively studied the fascinating flow dynamics of highly viscous core flow confined between low viscosity sheath flows in microchannels in a wide range of core/sheath flow ratios, viscosity ratios and diffusion coefficient ratios. These studies have demonstrated the formation of a highly stable ‘viscous thread’ at high Péclet numbers with a smooth interface formed between the core/sheath flows^23^. In contrast, applying moderate Péclet numbers is associated with some periodical oscillations at specific distances from the entrance of the core flow channel, where thread experiences ‘diffusive instabilities’ in the form of diffusive coiling. Applying low Péclet numbers leads to ‘ultra-diffusive instabilities’ where the core and sheath flows are mixed very quickly^23^. Also, it is shown that the combination of high sheath flow rates and low core/sheath viscosity ratios is associated with rolling up thin filaments of ‘viscous thread’, leading to ‘inertial instabilities’^23^.

In addition, deceleration of ‘viscous threads’ by means of diverging of the focusing channel is shown to induce ‘viscous buckling instabilities’, leading to formation of periodic folding threads^21, 24, 25^, similar to the case of a viscous liquid falling on a solid surface^26^. Furthermore, offsetting the viscous core flow from the middle of the focusing channel by means of applying asymmetric sheath flow rates is shown to induce sinuous perturbations in the direction transverse to the flow, which eventually leads to disintegration of the ‘viscous thread’ into isolated ‘viscous swirls’^22^.

In this work, we study the dynamics of a highly viscous core flow made of glycerol confined by sheath flows made of water within a microfluidic flow focusing system. The highly viscous core flow is tapered by sheath flows and eventually forms a thin ‘viscous thread’ along the middle of the channel. We have studied the dynamic characteristics of tapered core flow under various combinations of core-sheath flow rates. Our studies indicate that the dynamics of tapered core structures is greatly affected by the magnitude of sheath flow rate. At low sheath flow rates, the tapered core remains very stable, whereas at high sheath flow rates local instabilities are induced at the interface of core-sheath flows. Depending on the intensity of such local instabilities and the length of the core (both determined by the magnitude of sheath flow rate), the core structure can experience stable, disturbed, broken or oscillated regimes. A set of dimensionless numbers have been used to analyse the flow conditions required for the formation of highly dynamic ‘broken core’ and ‘oscillating core’ regimes, which to best of our knowledge have not been investigated before.

**Figure 1:**
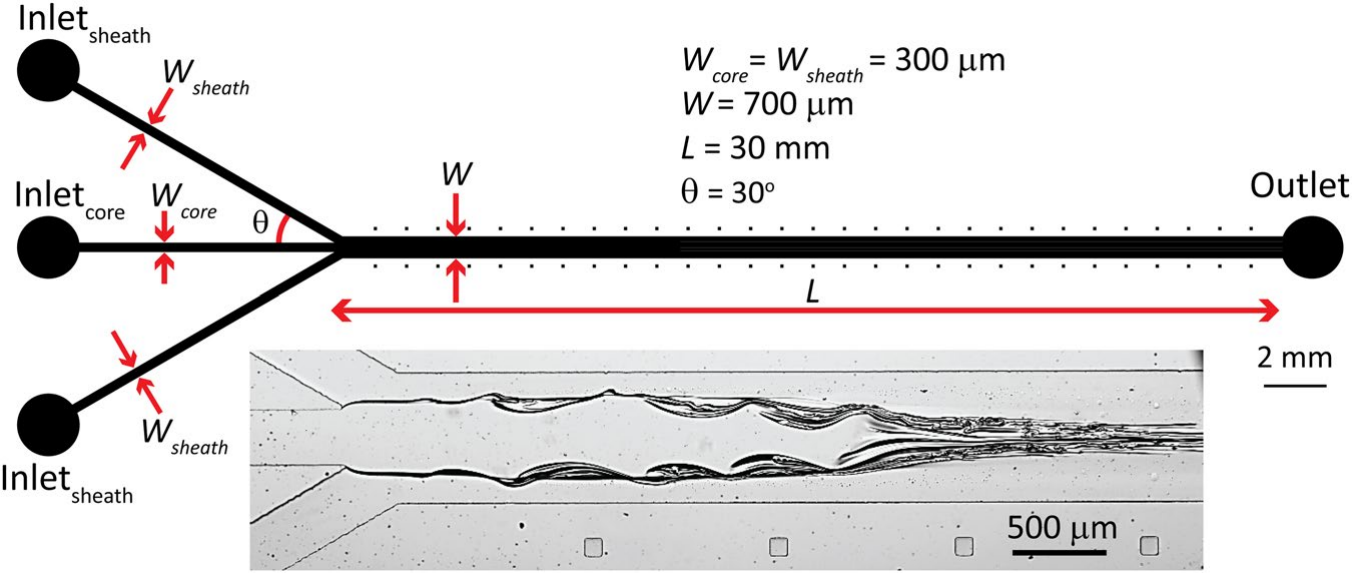
Schematics of the microfluidic flow focusing system. Inset shows the formation of the glycerol core flow between water sheath flows.

The experimental setup consists of a microfluidic flow focusing device for producing core/sheath flows of glycerol/water, two syringe pumps (Harvard 2000, Harvard PicoPlus) for introducing glycerol and water into the device, and a high-speed camera (Phantom Miro 310, 1000 fps) attached to an inverted microscope (Nikon Eclipse Ti) for visualisation of the core/sheath flow dynamics in real-time. Figure 1 presents the schematic of the microfluidic flow focusing device.

## Results

### Flow map

**Figure 2:**
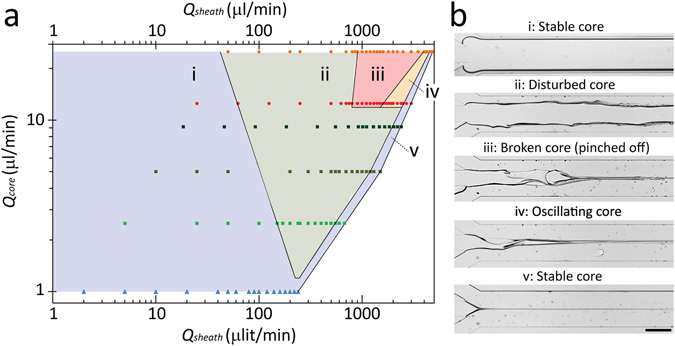
(**a**) Flow map of stable, disturbed, broken (pinched-off), and oscillating core regimes with respect to the flow rates of core and sheath flows, and (**b**) Snapshot images of corresponding flow regimes at *Q*_*core*_ = 12.5 µl/min: (i) ‘stable core’, *Q*_*sheath*_ = 62.5 µl/min, (ii) ‘disturbed core’, *Q*_*sheath*_ = 625 µl/min, (iii) ‘broken core’, *Q*_*sheath*_ = 1200 µl/min, (iv) ‘oscillating core’, *Q*_*sheath*_ = 1700 µl/min, (v) ‘stable core’, *Q*_*sheath*_ = 3000 µl/min.

First, we examine the core structure under various combinations of core and sheath flow rates applied into the flow focusing system. Six groups of experiments are conducted by setting the core flow rate to *Q*_*core*_ = 1, 2.5, 5, 9, 12.5 and 25 µl/min. For each group, the sheath flow rate starts from *Q*_*sheath*_ = 2*Q*_*core*_ and is increased until the core flow is tapered just at the entrance of the flow focusing channel. This enables us to obtain the flow map of the system, as presented in Fig. 2. Using this flow map, the dynamics of highly viscous core flow can be classified into four regimes, including ‘stable core’, ‘disturbed core’, ‘broken core’ and ‘oscillating core’, as summarised below:

(i) ‘Stable core’ regime refers to conditions that the core structure is highly stable, and a very smooth interface is formed between the neighbouring core-sheath flows. This regime (shaded in blue) occurs at either low (Fig. 2b-i) or very high (Fig. 2b-v) sheath flow rates. In both cases, the perturbations induced at the interface of core-sheath flows are decayed.
(ii) ‘Disturbed core’ regime refers to conditions that instability patterns are consistently induced at the interface of core-sheath flows. This regime (shaded in green) occurs at moderate sheath flow rates (Fig. 2b-ii).
(iii) ‘Broken core’ regime refers to conditions that severe instability patterns are induced at the interface of core-sheath flows. These instabilities severely deform the core structure and eventually break it into two parts (Fig. 2b-iii). This regime (shaded in pink) occurs when sheath flow rates are sufficiently high to induce severe instability patterns, and at the same time, the core is sufficiently long to be broken by such instabilities. This regime has not been reported for highly viscous core flows before.
(iv) ‘Oscillating core’ regime refers to conditions that the core structure is harmonically oscillated by asymmetric instability patterns induced at its two interfaces (Fig. 2b-iv). This regime (shaded in gold) occurs when sheath flow rates are sufficiently high to induce severe instability patterns but the core is not long enough to be broken by such asymmetric waves. Our work for the first time reports this regime for highly viscous core flows.

### Stable core regime

**Figure 3:**
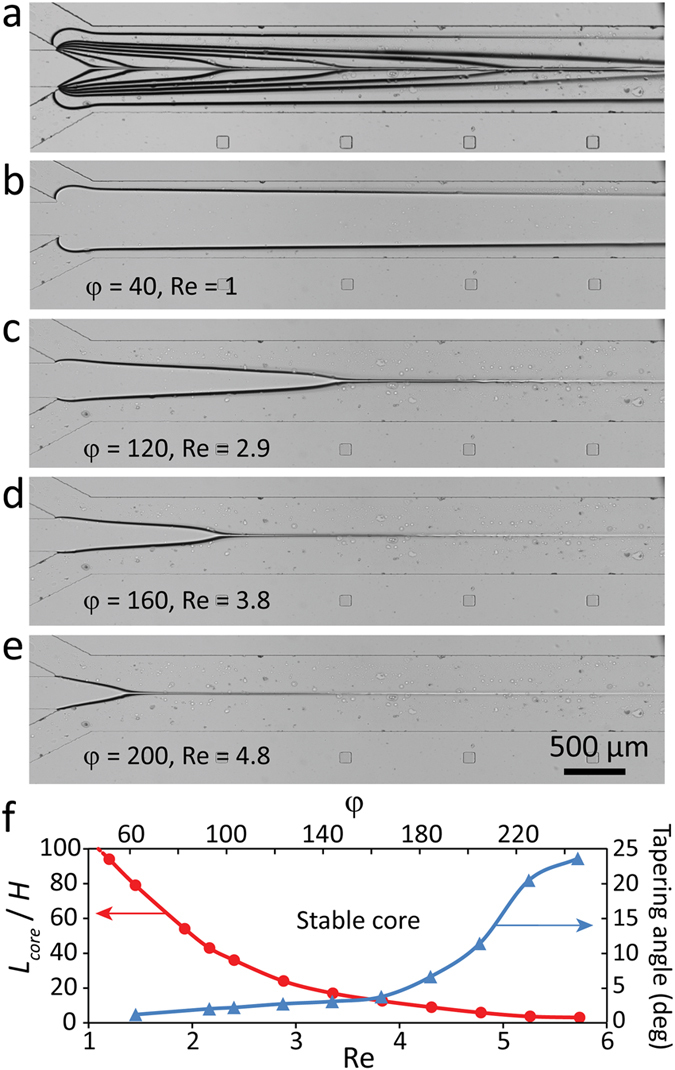
Formation of ‘stable core’ structures obtained by applying glycerol at 1 µl/min while applying water at varying flow rates of 2 to 240 µl/min: (**a**) Stacked images obtained at various sheath flow rates, (**b**–**e**) Individual images obtained at various sheath flows corresponding to Reynolds numbers of 1, 2.9, 3.8 and 4.8, showing the formation of ‘tapered core’ structures followed by ‘viscous thread’ at the downstream of the flow focusing channel, and (**f**) Variations of core length ratio against Reynolds number and sheath/core flow ratio.

Figure 3 presents ‘stable core’ structures obtained by applying glycerol core flow at 1 µl/min while water sheath flow at various rates ranging from 2 to 240 µl/min, presented as blue triangles in Fig. 2. We define a set of dimensionless numbers in order to analyse the results. This includes Reynolds number defined as Re = *ρ*_*sheath*_ *U*_*total*_ *H*/*µ*_*sheath*_, in which *U*_*total*_ = (*Q*_*core*_ + *Q*_*sheath*_)/(*WH*) is the average velocity of liquid through the flow focusing channel, sheath/core flow ratio defined as *φ* = *Q*_*sheath*_/*Q*_*core*_, core/sheath viscosity ratio defined as χ = *µ*_*core*_/*µ*_*sheath*_, and core length ratio defined as *L*_*core*_/*H*.

Figure 3a–e show the stacked and individual images of core-sheath structures formed at the beginning of the flow focusing channel at various Reynolds numbers and sheath/core flow ratios. The more detailed version of stable core structures is presented in Figure S1. The width of the core flow reduces by increasing the Reynolds number or sheath/core flow ratio, as expected in a microfluidic flow focusing system. For flow focusing systems with a core/sheath viscosity ratio of about one (which is the case for the majority of the works which use water-based solutions for both core and sheath flows), the core-sheath flows reach a balance at the entrance region of the channel, after which they form three parallel streams along the channel^3, 27^.

In contrast, here the core/sheath viscosity ratio is 210, and the highly viscous core flow is tapered by sheath flows until forming a thin stream along the channel, which is referred to as ‘viscous thread’ in the literature^21^. The tapering of the core can be attributed to the massive velocity gradient and consequently shear stress levels at the interface of the core-sheath flows, as predicted by our numerical simulations (Figure S2). Increasing the Reynolds number increases the magnitude of shear stress at the interface of core-sheath flows, and consequently accelerates the tapering of the core. This trend can be clearly seen in Fig. 3f, which shows the core tapering angle and core length ratio with respect to Reynolds number and sheath/core flow ratio, according to which, the core length ratio can be expressed as *L*_*core*_/*H* = 147 Re^−2.5^, whereas the core tapering angle can be calculated as *θ* = 0.02 Re ^4.05^.

### Disturbed core regime

**Figure 4:**
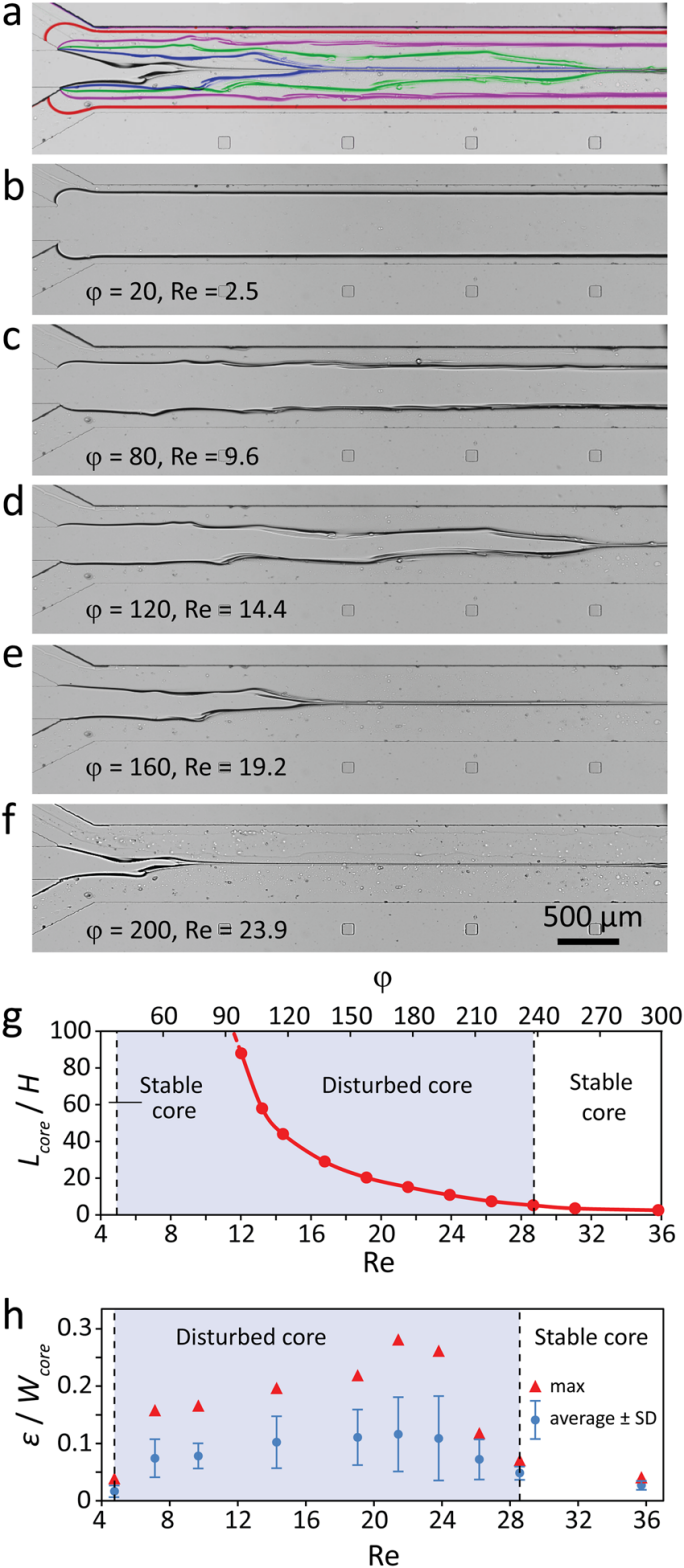
Formation of ‘disturbed core’ structures obtained by applying glycerol at 5 µl/min while applying water at varying flow rates of 10 to 1200 µl/min: (**a**) Stacked images obtained at various sheath flow rates, (**b**–**f**) Individual images obtained at various sheath flows corresponding to Reynolds numbers of 2.5, 9.6, 14.4, 19.2 and 23.9, showing the transition from ‘stable core’ to ‘disturbed core’ regime associated with intensified ‘interfacial waves’ at the interface of core-sheath flows, (**g**) Variations of core length ratio against Reynolds number and sheath/core flow ratio, and (**h**) Variations of core roughness ratio at the interface of core-sheath flows induced by ‘interfacial waves’ against Reynolds number.

Figure 4 presents ‘disturbed core’ structures obtained by applying glycerol at 5 µl/min while water at various flow rates ranging from 10 to 1200 µl/min, presented as green squares (third row from the bottom) in Fig. 2. Figure 4a–f show the stacked and individual images of core-sheath structures formed at the beginning of the flow focusing channel at various Reynolds numbers and sheath/core flow ratios, with more details presented in Figure S3.

‘Stable core’ regime is observed for Reynolds numbers smaller than 4.9. Increasing the Reynolds numbers within the range of 4.9 to 28.7 leads to ‘disturbed core’ regime, during which instability patterns are induced at the interface of core-sheath flows (Fig. 4c + Supplementary Movie 1). Such instabilities are referred to as ‘instability waves’^12^ or ‘interfacial waves’^13^ in the literature. ‘Interfacial waves’ are asymmetric with respect to the core and form multiple throughs and crests along the interface. Thin filaments of highly viscous core flow shed along the crests^28^. These filaments which move along the interface, and eventually merge with the ‘viscous thread’ formed at the tip of the tapered core flow. Increasing the Reynolds number within this range intensifies the ‘interfacial waves’, and leads to severe deformation of core structure (Fig. 4d + Supplementary Movie 2). Interestingly, the base and the tip of the tapered core seem to be mechanically robust and do not deform. As a result, the core behaves as a ‘simply supported beam’ and experiences the highest deformations along its middle regions. At Reynolds numbers larger than 28.7, the highly viscous core is so short that the ‘interfacial waves’ do not develop at its interface (Figure S3). In this case, a ‘stable core’ is formed, similar to the conditions occurring at low Reynolds numbers. Similar trends are observed when setting the core flow rate to 2.5 µl/min, as presented in Figure S4.

Our steady state numerical simulations do not predict the formation of ‘interfacial waves’ but clearly show the increased shear stress levels at the interface of the highly viscous core flow, which induce such instabilities (Figure S5). Comprehensive numerical simulations have predicted the formation of pearl and mushroom instabilities in the presence of highly viscous sheath flows^14, 29^. In these works, the flow variables (velocity, pressure, concentration of core and sheath flows and viscosity) are composed of steady state and perturbation components, and the Navier-Stokes equations are solved in transient mode. At low Reynolds numbers, the perturbations are decayed in consequent iterations, whereas at high Reynolds numbers, the perturbations are amplified and produce the transient ‘interfacial waves’ which lead to pearl and mushroom instabilities^14, 29^.

In practice, such perturbations can be induced due to any mismatch between the two sheath flows, caused by unequal sheath flow rates or fabrication deficiencies. In our work, water sheath flows are applied via two separate inlet ports (Fig. 1). These ports are infused by two syringes that are mounted onto the same syringe pump. To minimise any unwanted flow mismatch between the two water inlets, we used a microfluidic flow focusing system, in which water is applied through the same inlet port before branching into two inlet channels. Similar instability patterns are observed using this modified device.

Figure 4g shows the variations of core length ratio against Reynolds number and sheath/core flow ratio, according to which the core length ratio can be expressed as *L*_*core*_/*H* = 33500 Re^−2.5^. The instability of ‘interfacial waves’ is quantified using core roughness ratio, defined as *ε*/*W*_*core*_, in which *W*_*core*_ is the width of the core flow inlet channel, and *ε* is the distance between the interface and the virtual stable core formed, as detailed in Figure S6. Figure 4h presents the variations of maximum and average core roughness ratio with respect to the Reynolds number. This graph clearly shows the increase of core roughness ratio for the core structures falling under the ‘disturbed core’ regime (shaded in blue) with the highest *ε*_*max*_/*W* = 0.27 obtained at Re = 21.5.

### Broken core (pinched-off) regime

**Figure 5:**
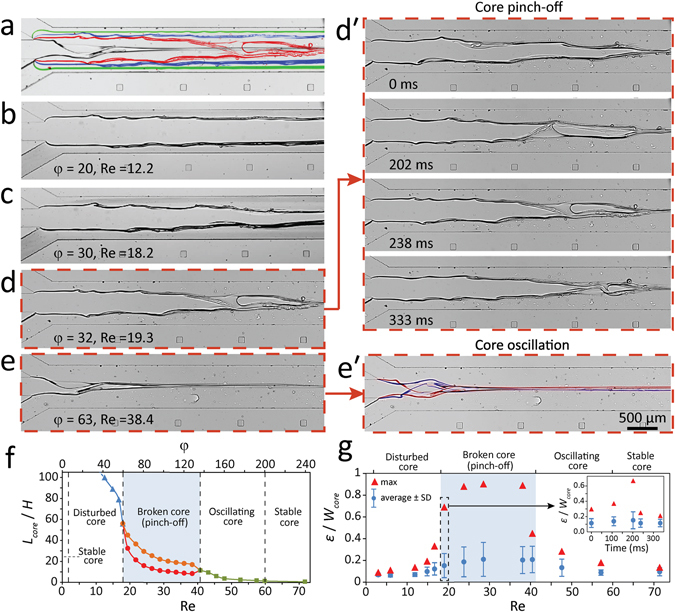
Formation of ‘broken core’ (pinched-off) and ‘oscillating core’ structures obtained by applying glycerol at 12.5 µl/min while applying water at varying flow rates of 25 to 3000 µl/min: (**a**) Stacked images obtained at various sheath flow rates, (**b**–**e**) Individual images obtained at various sheath flows corresponding to Reynolds numbers of 12.2, 18.2, 19.3, and 38.4, showing the transition from ‘disturbed core’ to ‘broken core’ and ‘oscillating core’ regimes, (**dʹ**) Four stages of ‘broken core’ regime, involving core halting, expansion of waves, pinch-off and recovery, (**eʹ**) Stacked images of ‘oscillating core’, (**f**) Variations of core length ratio against Reynolds number and sheath/core flow ratio, and (**g**) Variations of core roughness ratio at the interface of core-sheath flows against Reynolds number.

Figure 5 presents ‘broken core’ structures obtained by applying glycerol at 12.5 µl/min while water at various flow rates ranging from 25 to 3000 µl/min. These structures have been presented as red circles (fifth row from the bottom) in Fig. 2. Figure 5a–e show the stacked and individual images of core-sheath structures formed at the beginning of the flow focusing channel obtained at various Reynolds numbers, with more details shown in Figure S7.

The conditions described here exhibits all four flow regimes presented in Fig. 2. This involves ‘stable core’ regime with characteristic smooth interfaces for Reynolds numbers smaller than 3.3. This is followed by ‘disturbed core’ regime with characteristic ‘interfacial waves’ induced at the interface of core-sheath flows for Reynolds numbers ranging from 3.3 to 18.2.

Surprisingly, for the Reynolds numbers within the range of 18.2 to 40.8, we observed a completely new phenomenon, in which the core cannot maintain its continuity and is broken by intensified ‘interfacial waves’. This phenomenon, which we coin it as ‘core pinch-off’, is presented in Supplementary Movies 3 to 5. Droplet pinch-off is a well-established technique that is used for the continuous generation of microscale droplets in microfluidics^30–33^. While this technique is well characterised for a pair of immiscible liquids (e.g. water-oil or water-air), it is not characterised for a pair of miscible liquids such as glycerol and water investigated here.

Our observations indicate that this dynamic phenomenon consists of four stages, as captured in Figure 5d′. The first stage involves ‘core halting’ in which the core does not advance along the flow focusing channel, very similar to the conditions presented in Fig. 4d. The second stage involves ‘expansion of waves’ in which strong ‘interfacial waves’ are induced at the interface of core-sheath flows. The throughs caused by these waves can be as large as the width of the core. The third stage involves ‘core pinch-off’ during which the core is broken by a large through. The fourth stage involves ‘core recovery’ during which the broken core advances along the channel. Our simulations clearly show the increased shear stress levels induced at the interface of core-sheath flows but are unable to model the intensified ‘interfacial waves’, which break the core structure (Figure S8).

Increasing the Reynolds numbers within the range of 40.8 to 59.8 exhibits a new dynamic phenomenon, in which the core flow is not long enough to be broken by ‘interfacial waves’ but is oscillated harmonically under the influence of asymmetric waves, as presented in the stacked Fig. 5e′. This phenomenon, which we coin it as ‘core oscillation’, is presented in Supplementary Movie 6. The oscillating flow patterns observed here are very similar to ‘von Karman’ vortices produced at the downstream of circular structures^34^. For Reynolds numbers larger than 59.8, ‘stable core’ regime is observed again, as the core is very short.

Figure 5f presents the variation of core length ratio with respect to Reynolds number and sheath/core flow ratio. For the case of ‘broken core’ regime (shaded in blue), the core structure varies between minimum and maximum values corresponding to post- and pre- pinch-off conditions. By assuming *L*_*core*_ as the average length of the ‘broken core’, the core length ratio can be expressed as *L*_*core*_/*H* = 76000 Re^−2.5^ across the entire core regimes described here.

Figure 5g presents the variations of maximum and average roughness ratio against Reynolds number. The core structures falling in the ‘broken core’ regime (shaded in blue) exhibit the highest core roughness ratios with the highest value of *ε*_*max*_/*W*_*core*_ = 0.9 obtained at Re = 28.6. At the point of core pinch-off, the maximum roughness (corresponding to the deepest through produced at the interface) reaches the local width of the core. As described above, the ‘broken core’ regime involves four dynamic stages (core halting, expansion of waves, pinch-off and recovery), during which the roughness values change dynamically, as presented in Fig. 5g-inset.

Further experiments are conducted to investigate whether the induction of severe ‘interfacial waves’ is influenced by the junction angle between the core and sheath flow channels. In doing so, glycerol is applied through the central and one of the side inlet channels at a total flow rate of 25 µl/min while water is applied through the third inlet channel at various flow rates ranging from 50 to 1250 µl/min. In this case, a single interface is formed between the neighbouring glycerol and water flows (Figure S9). Experiments indicate three distinct flow regimes, including: (i) ‘stable interface’ at Reynolds numbers smaller than 3.6 (similar to ‘stable core’ regime), (ii) ‘disturbed interface’ caused by weak to moderate ‘interfacial waves’ at Reynolds numbers ranging from 3.6 to 24.4 (similar to ‘disturbed core’ regime), and (iii) ‘highly disturbed interface’ caused by severe ‘interfacial waves’ at Reynolds numbers larger than 24.4 (similar to the ‘broken core’ regime). These severe waves are very transient in nature and can expand up to 80% of the channel, as presented in Figure S9 + Supplementary Movie 7. Similar instability patterns are obtained by applying water and glycerol flows into a T-mixer. These experiments indicate that the instability patterns, which cause the ‘disturbed core’ and ‘broken core’ regimes in our flow focusing system, are not influenced by the junction angle between the core and sheath flow channel, and can be induced when the flow Reynolds number and the core length are large enough to amplify the ‘interfacial waves’.

**Figure 6:**
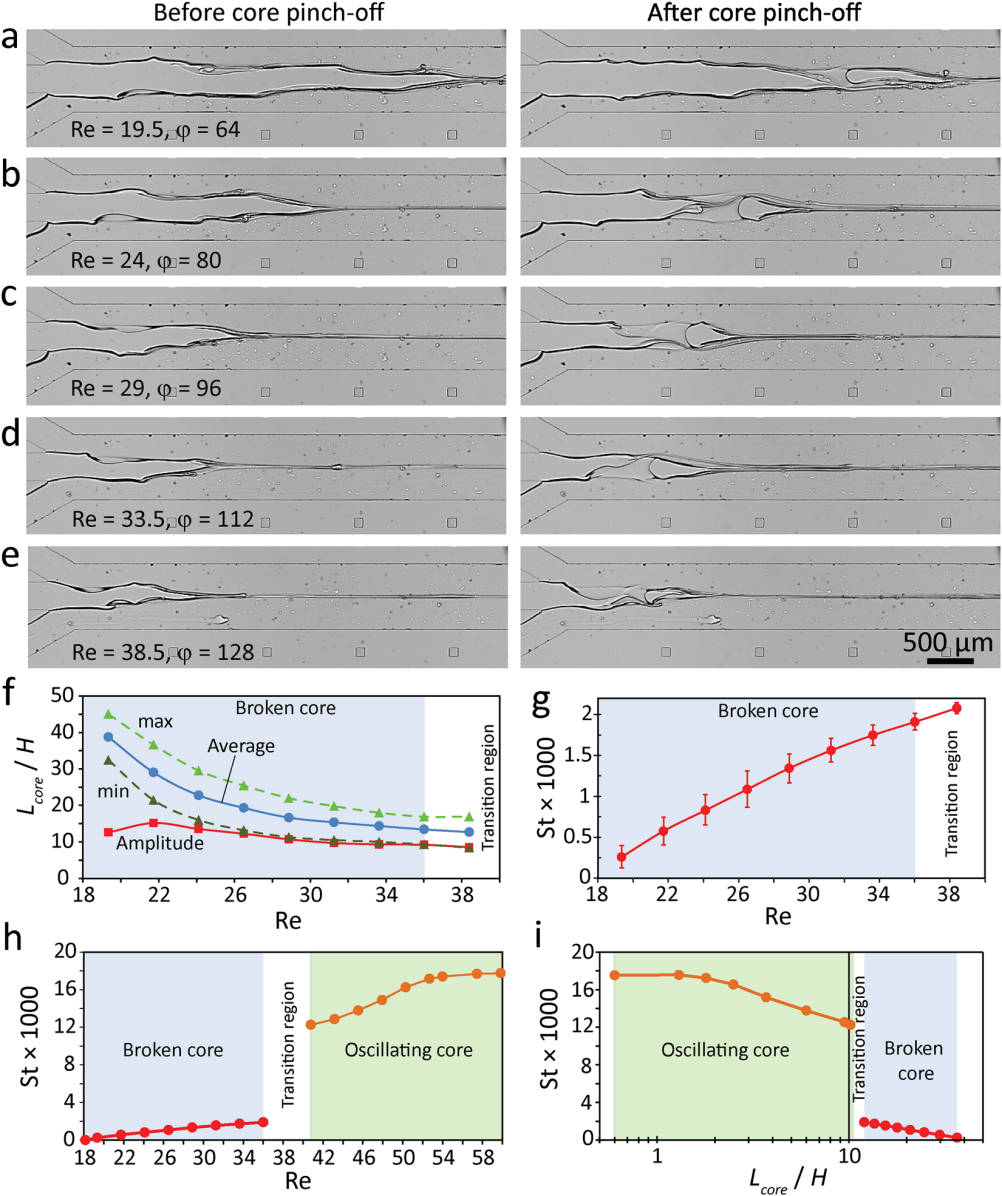
Dynamics of highly viscous core undergoing ‘broken core’ and ‘oscillating core’ regimes: (**a**–**e**) Extent of the core before/after pinch-off at various Reynolds numbers of 19.5, 24, 29, 33.5 and 38.5 (**f**) Variations of core length ratio against Reynolds number, (**g**) Variations of core Strouhal number against Reynolds numbers for ‘broken core’ regime, (**h**) Variations of core Strouhal number against Reynolds numbers across ‘broken core’ and ‘oscillating core’ regimes, and (**i**) Variations of core Strouhal number against core length ratio across ‘broken core’ and ‘oscillating core’ regimes.

We conducted further experiments to explore the dynamic nature of highly viscous core flow when undergoing ‘broken core’ and ‘oscillating core’ regimes. Figure 6a–e present the extent of the highly viscous core before and after the pinch-off process. Results are obtained by applying glycerol at 12.5 µl/min while water at various flow rates of 800, 1000, 1200, 1400 and 1600 µl/min, corresponding to Reynolds numbers of 19.5, 24, 29, 33.5 and 38.5 (Supplementary Movies 3 to 5).

Figure 6f shows the variations of maximum and minimum core length ratios (corresponding to per- and post- pinch-off conditions) with respect to Reynolds number, using which the average and amplitude of core displacement have been calculated. Using these graphs, the maximum, average and minimum core length ratios can be expressed as 5023/Re^−1.6^, 7474/Re^−1.795^ and 23097/Re^−2.24^, respectively, whereas the amplitude of core displacement ratio can be described as 11.2 ± 2.3 (Average ± SD). Our experiments indicate that towards the end of ‘broken core’ regime, the core experiences a transition regime in which the core pinch-off is not consistent (shaded in white).

In order to quantify the frequency of core pinch-off, we define the core Strohaul number as St = *f H*/*U*_*total*_, in which *f* is the frequency, and *U*_*total*_ is the average velocity of liquid through the flow focusing channel, as has been used for calculating Reynolds number. This is similar to the concept used in ref. 13 to non-dimensionalise the frequency of mushroom and pearl waves induced at the interface of low viscosity core. Figure 6g shows the variations of core Strouhal number against Reynolds number for ‘broken core’ regime, across which the Strouhal number increases almost linearly that can be expressed as St = 10^−4^ × (Re–15).

Figure 6h shows the variations of core Strouhal number against Reynolds number across ‘broken core’ and ‘oscillating core’ regimes. This graph clearly shown the sudden increase of core Strouhal number across the transition region. This is because the breaking of the core involves the four dynamic stages, including the core halting, expansion of waves, pinch-off and recovery, as discussed in Fig. 5d′, whereas the oscillation of the core is simpler and only involves the bending of the core, as shown in Fig. 5e′. Entering the ‘oscillating core’ regime, Strouhal number increases parabolically that can be best expressed as St = 5.2 × 10^−4^ Re^1.46^, whereas towards the end of ‘oscillating regime’ Strouhal number becomes saturated.

To further understand the dynamics of core across the ‘broken core’ and ‘oscillating core’ regimes, we investigate the variations of core Strouhal number against core length ratio, as presented in Fig. 6i. Shortening of the core across the ‘broken core’ regime increases the core Strohaul number, which can be expressed as St = 0.0496 (*L*_*core*_/*H*)^−1.286^. The core Strouhal number experiences a sudden increase across the transition region. Likewise, shortening of the core across the ‘oscillating core’ regime raises the core Strohaul number, which can be best expressed as St = 0.0011 + 0.0043/(1 + (0.0477 *L*_*core*_/*H*)^1.4^).

### Mapping the core configuration under various regimes

**Figure 7:**
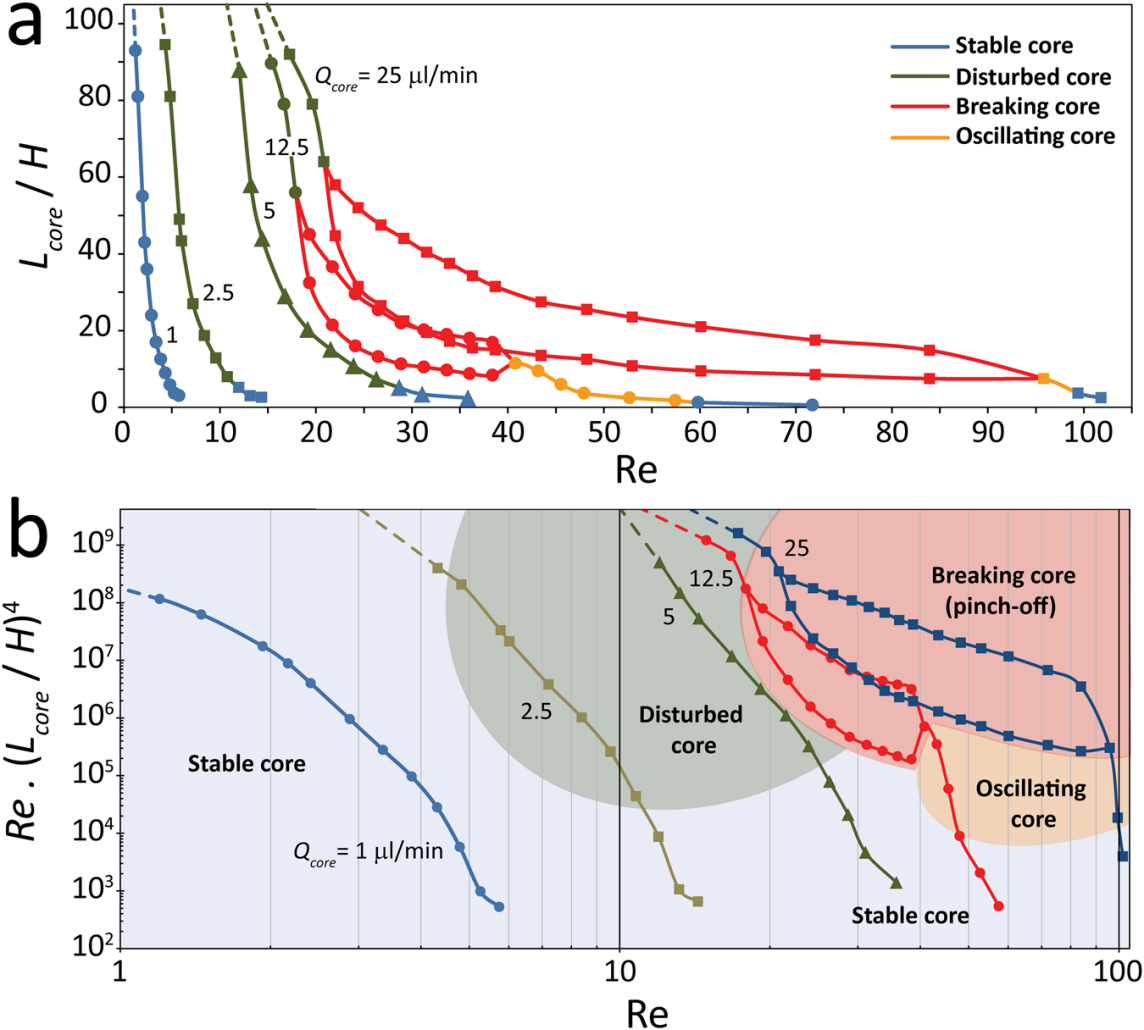
(**a**) Variation of dimensionless core length against Reynolds number under various combinations of core and sheath flow rates, as presented in Figs 3 to 6, and (**b**) Variation of Re. (*L*_core_/*H*)^4^ (representing the deflection of glycerol core flow under dynamic loads) against Reynolds number.

Figure 7a illustrates the variations of core length ratio against Reynolds number obtained for various core flow rates of 1, 2.5, 5, 12.5 and 25 µl/min, as described in Figs 3 to 5. In order to describe the dynamics of the highly viscous core, we have divided the represented core length ratio curves into blue, green, red and golden segments corresponding to stable, disturbed, breaking and oscillating core regimes occurring in each segment.

Increasing the Reynolds number increases the shear stress levels at the interface of core-sheath flows, as predicted in Figures S2, S5 and S8. This in turn intensifies the ‘interfacial waves’ induced at the interface, as quantified by core roughness ratio presented in Figs 4h and 5g, depending on which the highly viscous core flow can experience various stability regimes. However, a close look at Fig. 7a reveals that various core regimes can be produced at the same Reynolds number. For example, applying a Reynolds number of 25 leads to ‘disturbed core’ and ‘broken core’ regimes at the core flow rates of 5 and 12.5 µl/min, respectively. Likewise, applying a Reynolds number of 50 leads to ‘oscillating core’ and ‘broken core’ regimes at the core flow rates of 12.5 and 25 µl/min, respectively. This suggests that the dynamics of highly viscous core flow is not solely governed by Reynolds number.

To better describe the dynamics of the highly viscous core, we define the dimensionless core deformation as Δ = Re (*L*_*core*_/*H*)^4^. This definition originates from the following equation, Δ_max_ = *ωL* ^4^/48*EI*, which describes the maximum deflection of a simply supported beam under distributed load, in which Δ is deflection, *ω* is the distributed load density, *L* is the length of the beam, *E* is the elastic modulus, and *I* is the bending moment inertia of the structure^35^. As discussed previously, the disturbed core behaves as a simply supported beam with the highest deformations occurring at its middle regions (Fig. 4d–f). In this regard, the distributed load caused by’interfacial waves’ is represented by Re, and the length of the beam is represented by *L*_*core*_/*H*.

Figure 7b presents the variations of dimensionless core deformation with respect to Reynolds number for various core flow rates of 1, 2.5, 5, 12.5 and 25 µl/min. This figure enables us to better understand the conditions required to create stable, disturbed, broken and oscillating core regimes. At the top right of this chart lies the ‘breaking core’ regime (shaded in pink) at which both Re values (corresponding to the intensity of ‘interfacial waves’) and Re (*L*_*core*_/*H*)^4^ values (corresponding to the magnitude of core deformation) are high enough to break the core. Below this pink region lies the ‘oscillating core’ regime (shaded in gold) at which Re values are high enough to produce severe ‘interfacial waves’ disturb the core structure but Re (*L*_*core*_/*H*)^4^ values are not are high enough to break the core. At the left side of the pink region lies the ‘disturbed core’ regime at which Re values are not high enough to induce severe ‘interfacial waves’ but Re (*L*_*core*_/*H*)^4^ values are high enough to deform the core structure. Finally, at the bottom and left side of this chart lies the ‘stable core’ regime (shaded in blue), at which both Re and Re (*L*_*core*_/*H*)^4^ values are low and the perturbations induced along the core structure are decayed.

We conducted extended experiments to explore the dynamics of highly viscous core flow at the core/sheath viscosity ratio of 60, for which glycerol is diluted with water at a volume ratio of 9:2.4 (*µ*_*glycerol*_ = 0.06 Pa.s, *µ*_*water*_ = 0.001 Pa.s). Experiments are conducted at various core flow rates of 1, 5, 12.5 and 25 µl/min, as presented in Figures S10 to S13. Analysis of these results indicate the existence of stable, disturbed, broken and oscillating core regimes, similar to the core/sheath viscosity ratio of 210, as presented in Figure S14.

## Conclusion

In summary, we have investigated the dynamics of highly viscous core flows surrounded by low viscosity sheath flows in a microfluidic flow focusing channel. Our experiments indicate the formation of tapered core structures under the massive shear stress induced at the interface of core-sheath flows. Increasing the magnitude of sheath flow rate increases the magnitude of shear stress, and therefore shortens these core structures. More importantly, increase of sheath flow rate induces local instability patterns at the interface of core-sheath flows, which propagate in the form of ‘interfacial waves’. Such waves greatly impact the stability of the core structure, according to which the dynamics of the core structure can be classified into ‘stable core’, ‘disturbed core’, ‘broken core’ and ‘oscillating core’ regimes. The two important parameters, which determine the transition from one regime to another, are the sheath flow rate, represented by Reynolds number (Re), and the length of tapered core structure, represented by core length ratio (*L*/*H*). Our comprehensive experiments indicate that the highest deformation of the viscous core happens along its middle regions. In this essence, the highly viscous core can be regarded as a simply supported beam, which is consistently deformed by asymmetric ‘interfacial waves’ induced at the interface of core-sheath flows. Such deformations can be represented by Re (*L*/*H*)^4^ in which Re represents the distributed load generated by ‘interfacial waves’ over the core structure. Presenting the variations of Re (*L*/*H*)^4^ against Re enables us to map the dynamics of the highly viscous core, and predict the conditions required for the formation of each core regime. For example, this flow map shows that the ‘broken core’ regime occurs when Re and Re (*L*/*H*)^4^ values are both high. Using high-speed imaging, we have studied the dynamic characteristics of ‘broken core’ regime, and analysed the amplitude and frequency of core displacement within this regime.

Our work sheds a light on unexplored aspects of highly viscous core flows stratified by low viscosity sheath flows. The work can be extended by applying a pair of immiscible core-sheath flows (e.g. oil-water flows) to investigate the role of interfacial forces in the stability of the core. An extensive numerical analysis is also suggested to better understand the physics underlying the instability of the core when undergoing ‘broken core’ and ‘oscillating core’ regimes. Such instability patterns can be employed for enhancing the convective heat transfer at the entrance of the flow focusing channel, sorting particles, and selective transition of particles across the core-sheath interface.

## Methods

The device has three inlet channels for infusing glycerol core flow and water sheath flows (*W*_*core*_ = *W*_*sheath*_ = 300 µm). The length of inlet channels is set to 10 mm to ensure the inlet flows are fully developed before reaching the flow focusing channel. The junction angle between the core and sheath channels is set to 30° to ensure the microfluidic system can be accommodated along the width of a microscopic glass slide (*W* × *L* = 25 mm × 75 mm). All three inlet channels converge into a flow focusing channel (*W* × *L* = 700 µm × 30 mm). The height of the microfluidic device is *H* = 100 µm.

The microfluidic device is fabricated from polydimethylsiloxane (PDMS) using soft lithography techniques. A 1 mm thick glass slide and plasma treated in a Harrick plasma cleaner for 5 minutes and then attached to the PDMS channel. The assembly is then post baked at a 75 °C oven for 2 hours to permanently bond the glass slide to the PDMS moulded channel.

Glycerol is diluted with water at a volume ratio of 9:1 to produce a viscosity ratio of 210:1 (*µ*_*glycerol*_ = 0.210 Pa.s, *µ*_*water*_ = 0.001 Pa.s). Diluted glycerol is applied into the microfluidic device using a 5 ml syringe mounted onto a Harvard PicoPlus syringe pump while water is applied using two 20 ml syringes both mounted onto a Harvard 2000 syringe pump. The outlet of the microfluidic device is coupled to a collection beaker via a 0.06 inch Tygon® tube. Figure 1 inset shows the formation of a single central glycerol core flow focused between water sheath flows entering from both sides.

## Electronic supplementary material

Supplementary Information

Supplementary Movie 1

Supplementary Movie 2

Supplementary Movie 3

Supplementary Movie 4

Supplementary Movie 5

Supplementary Movie 6

Supplementary Movie 7
