# Supplementary material for: Dynamics of high viscosity contrast confluent microfluidic flows
Source: Sci Rep. 2017 Jul 19;7:5945. doi: 10.1038/s41598-017-06260-6 (PMC5517621; doi:10.1038/s41598-017-06260-6)
Supplement: Supplementary file 1 — Supplementary Information [file 41598_2017_6260_MOESM1_ESM.pdf]

Supplementary Information

Dynamics of high viscosity contrast confluent microfluidic flows

Michael E.Kurdzinski[1], Berrak Gol[1], Aaron Co Hee[1], Peter Thurgood[1], Jiu Yang Zhu[1], Phred Petersen[2], Arnan Mitchell[1,*], Khashayar Khoshmanesh[1,*]

[1]School of Engineering, RMIT University, Melbourne, Victoria, Australia

[2]School of Media and Communication, RMIT University, Melbourne, Victoria, Australia

* Corresponding authors: Khashayar.khoshmanesh@rmit.edu.au

Supplementary Information 1: Dynamics of core-sheath flows obtained at μ_{core}/μ_{sheath} = 210, Q_{core} = 1 μl/min

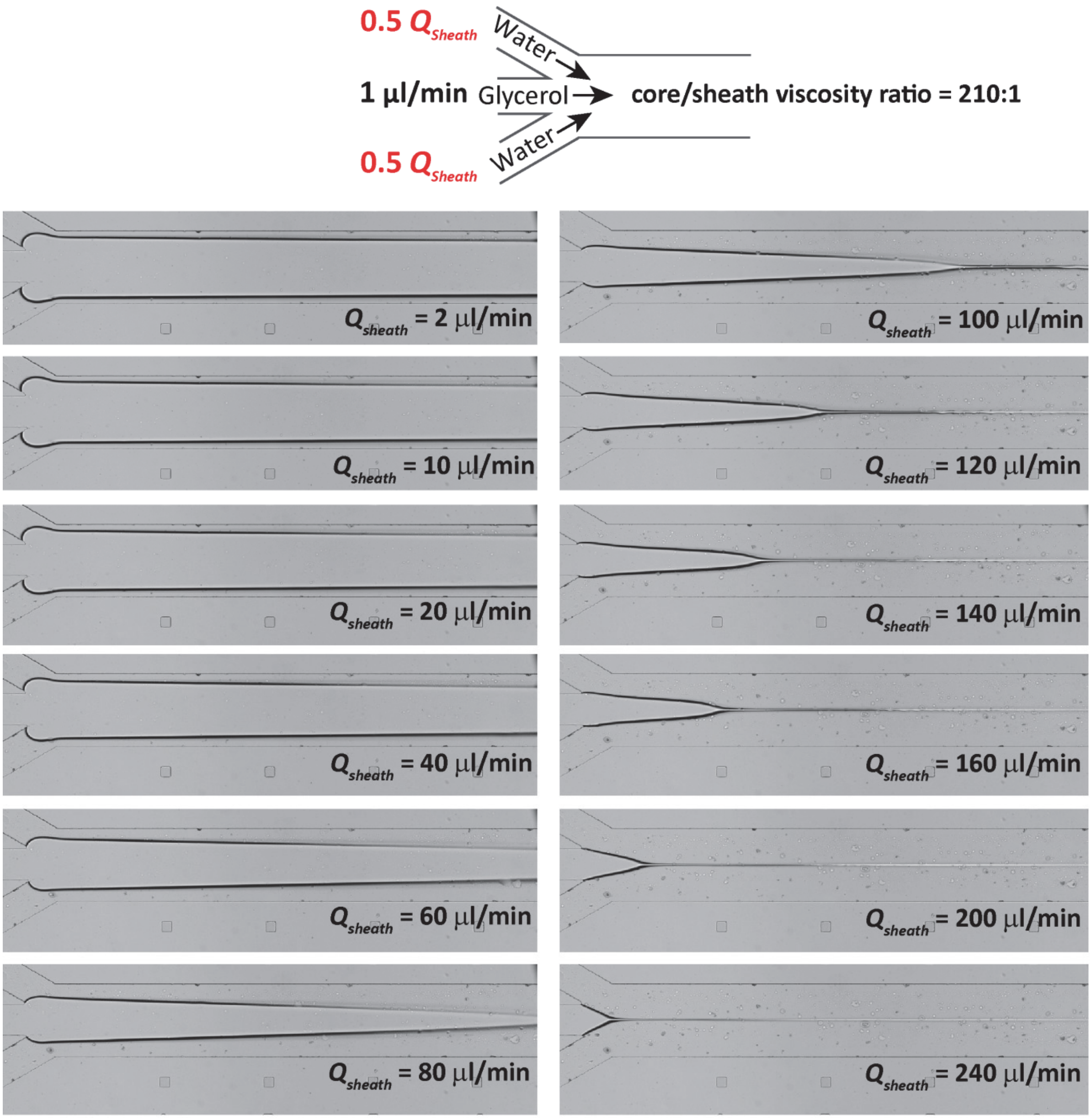

Figure S1. Dynamics of core-sheath flows under 'stable core' regime obtained by setting Q_{core} = 1 μl/min while Q_{sheath} = 2 to 240 μl/min.

Supplementary Information 2: Numerical simulation of core-sheath flows at μ_{core}/μ_{sheath} = 210, Q_{core} = 1 µl/min, Q_{sheath} = 120 µl/min

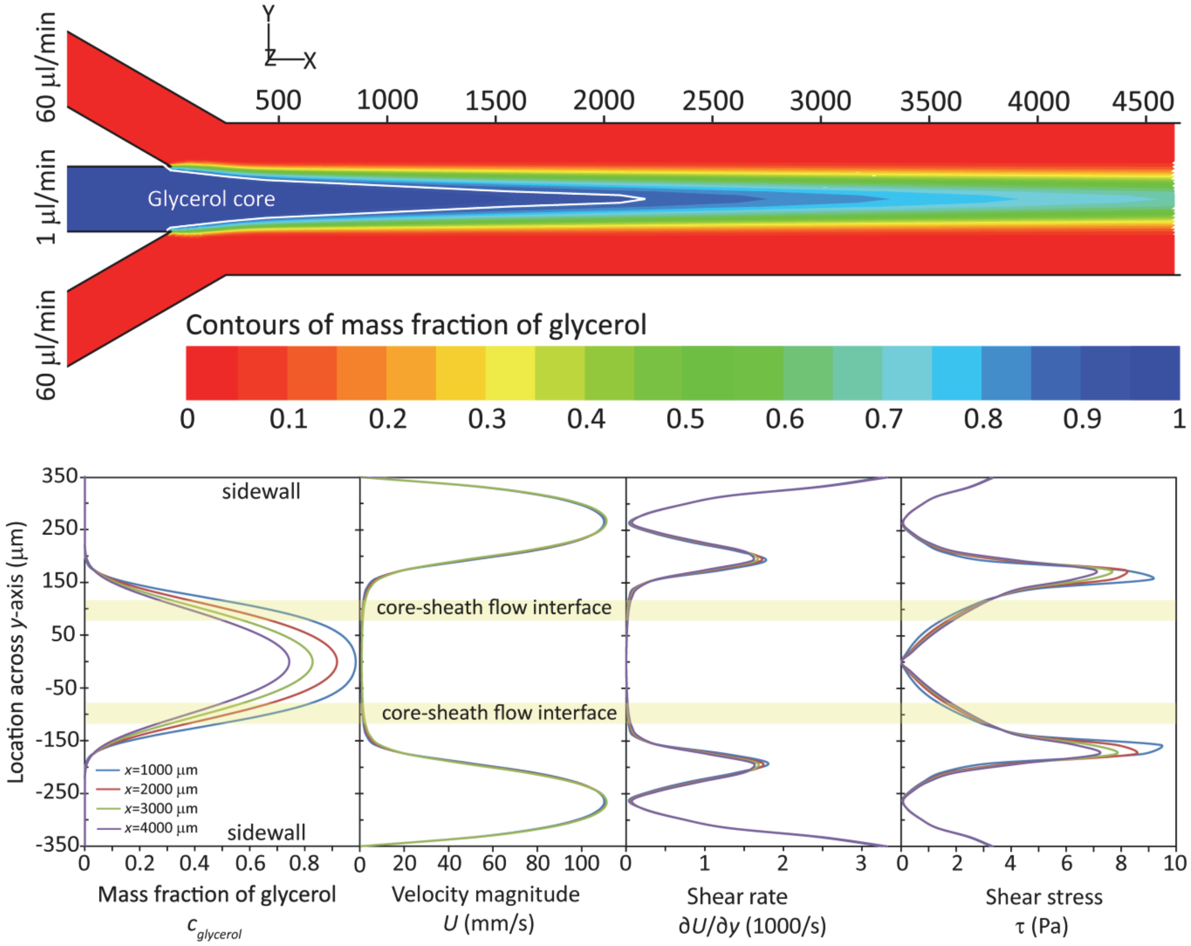

Figure S2. Numerical simulation of core-sheath flows obtained at μ_{core}/μ_{sheath} = 210, Q_{core} = 1 µl/min, Q_{sheath} = 120 µl/min (corresponding to 'stable core' regime). Results show the contours of glycerol mass fraction at the bottom surface of the channel, along with variations of glycerol mass fraction, velocity magnitude, velocity gradient (shear rate) and shear stress along the width of the flow focusing channel.

Supplementary Information 3: Dynamics of core-sheath flows obtained at $\mu_{core} / \mu_{sheath}$ = 210, Q_{core} = 5 µl/min

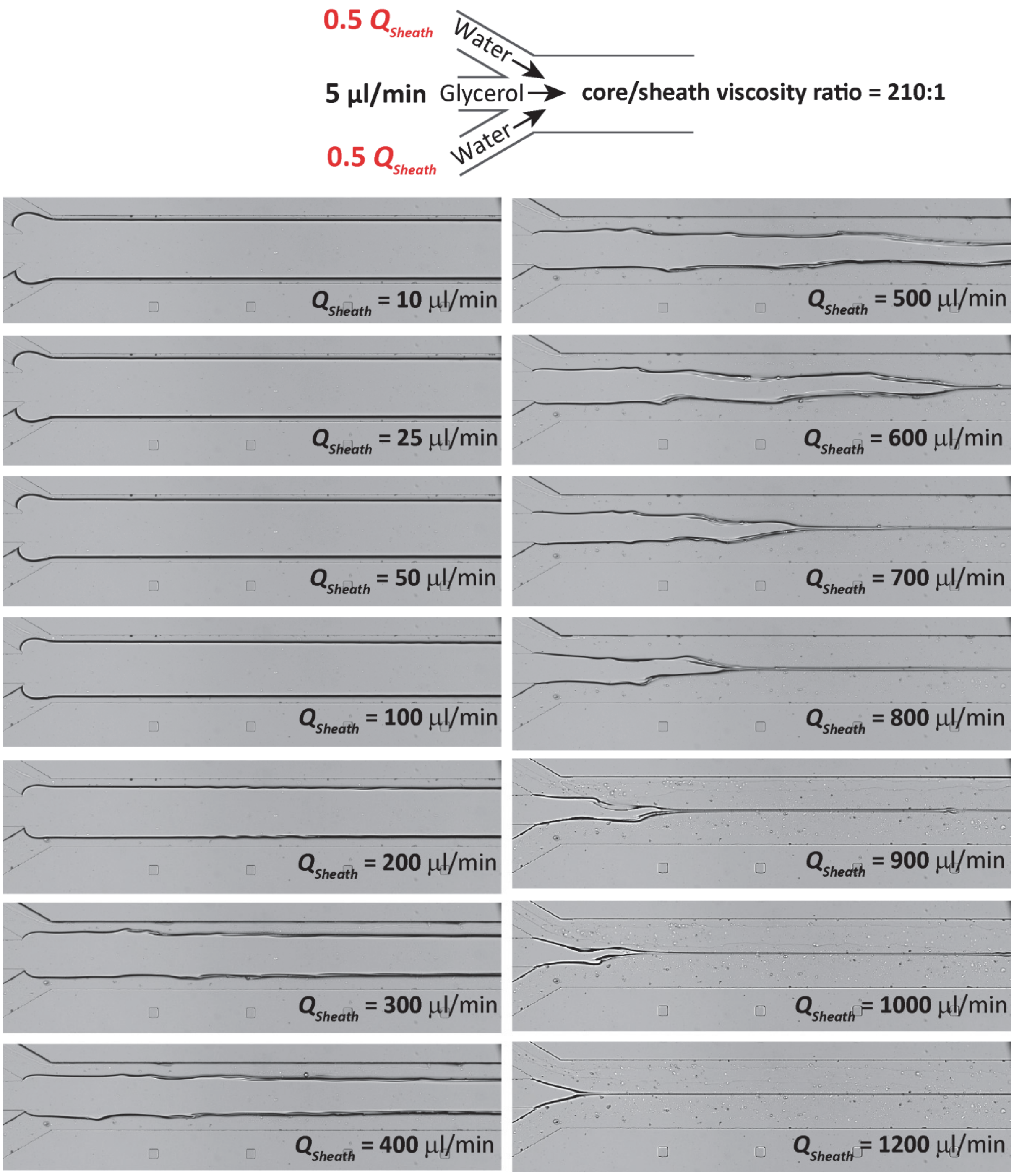

Figure S3. Dynamics of core-sheath flows under 'stable core' and 'disturbed core' regimes obtained by setting Q_{core} = 5 µl/min while Q_{sheath} =10 to 1200 µl/min.

Supplementary Information 4: Dynamics of core-sheath flows obtained at μ_{core}/μ_{sheath} = 210, Q_{core} = 2.5 µl/min

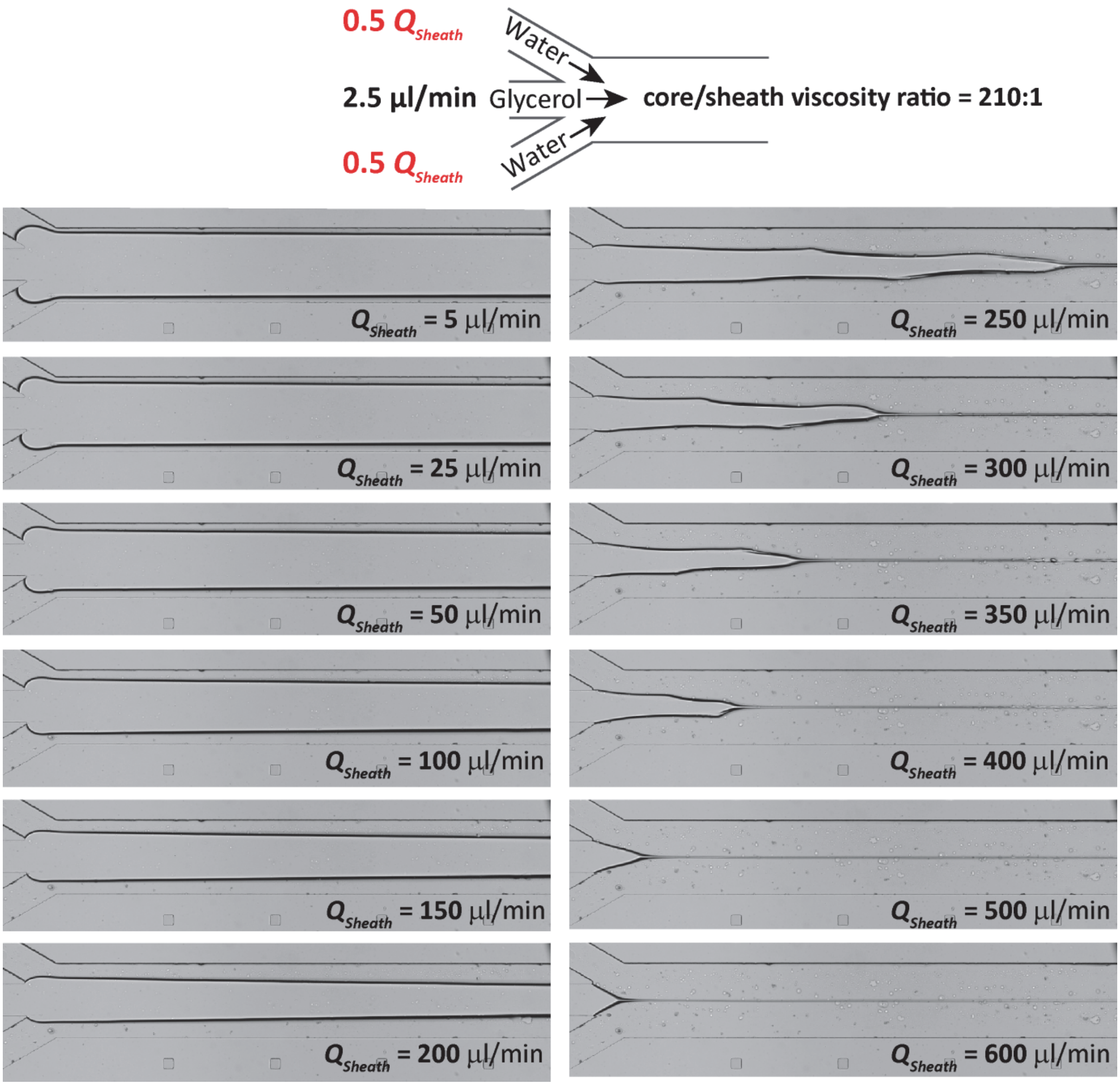

Figure S4. Dynamics of core-sheath flows under 'stable core' and 'disturbed core' regimes, obtained by setting Q_{core} = 2.5 µl/min while Q_{sheath} = 5 to 600 µl/min.

Supplementary Information 5: Numerical simulation of core-sheath flows at $\mu_{core}/\mu_{sheath} = 210$, $Q_{core} = 5$ µl/min, $Q_{sheath} = 600$ µl/min

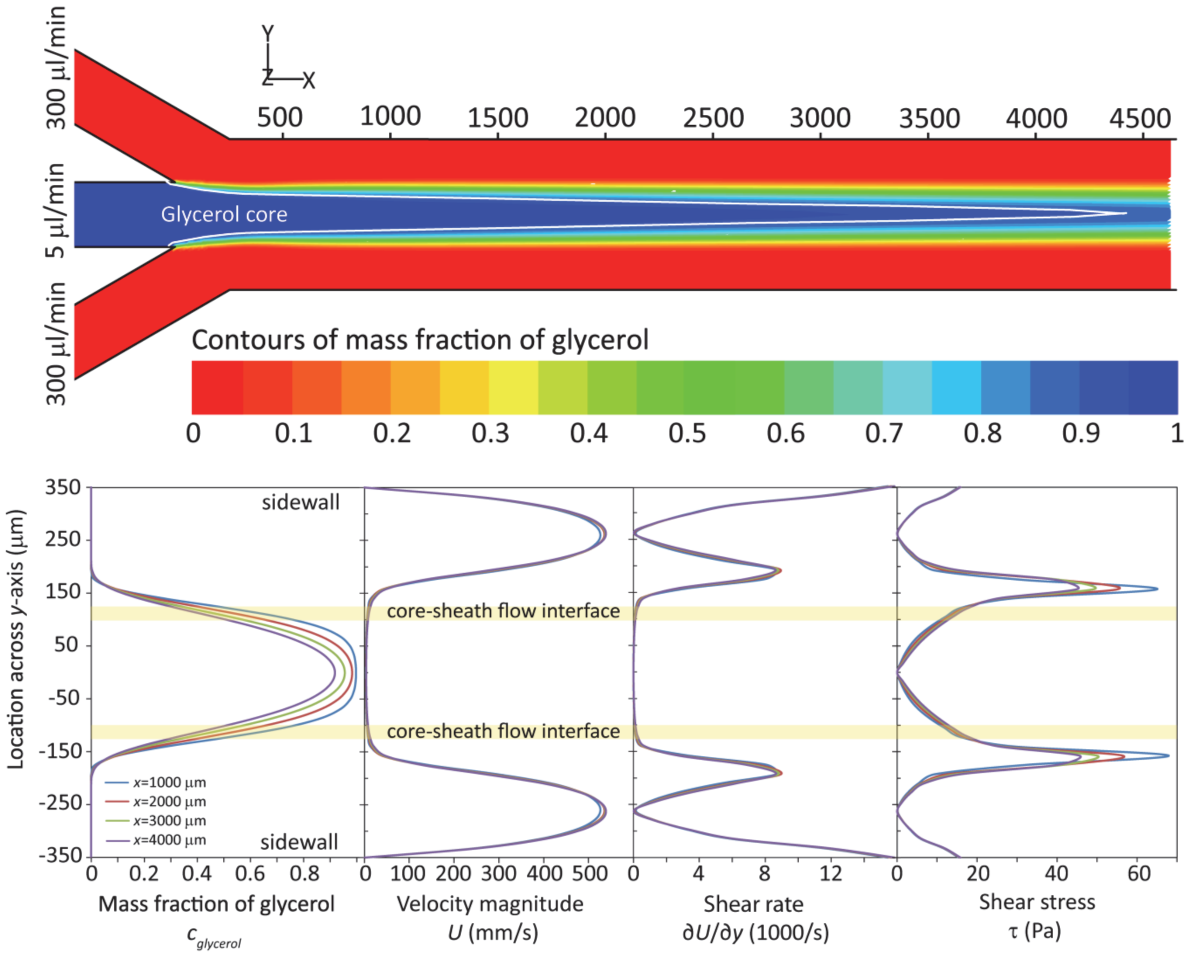

Figure S5. Numerical simulation of core-sheath flows obtained at $\mu_{core}/\mu_{sheath} = 210$, $Q_{core} = 5$ µl/min, $Q_{sheath} = 600$ µl/min (corresponding to 'disturbed core' regime). Results show the contours of glycerol mass fraction at the bottom surface of the channel, along with variations of glycerol mass fraction, velocity magnitude, velocity gradient (shear rate) and shear stress along the width of the flow focusing channel.

Supplementary Information 6: Measurement of roughness at the interface of core-sheath flows at μ_{core}/μ_{sheath} = 210, Q_{core} = 5 µl/min

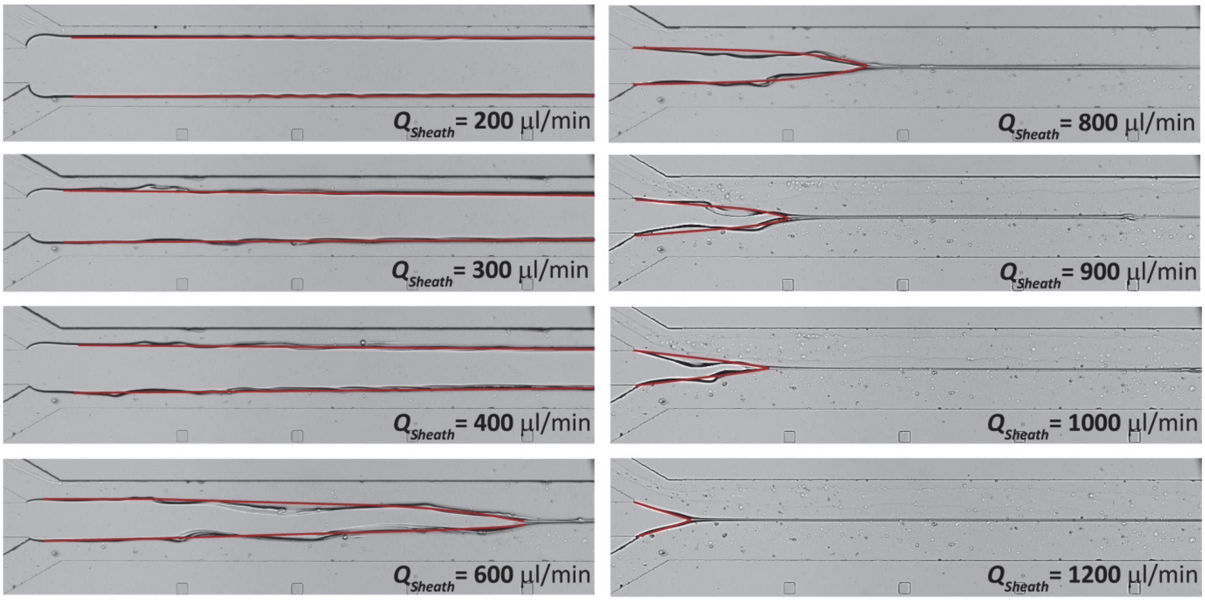

Figure S6: Measurement of roughness at the interface of core-sheath flows under 'stable core' and 'disturbed core' regimes, obtained by setting μ_{core}/μ_{sheath} = 210, Q_{core} = 5 µl/min and Q_{sheath} = 200 to 1200 µl/min. The red lines correspond to the virtual interface of stable core structures obtained under each case. The roughness is defined as the vertical distance between the virtual and real interfaces, and is calculated along 50 points on each side of the core-sheath interface using Matlab software.

Supplementary Information 7a: Dynamics of core-sheath flows obtained at $\mu_{core} / \mu_{sheath}$ = 210, Q_{core} = 12.5 µl/min

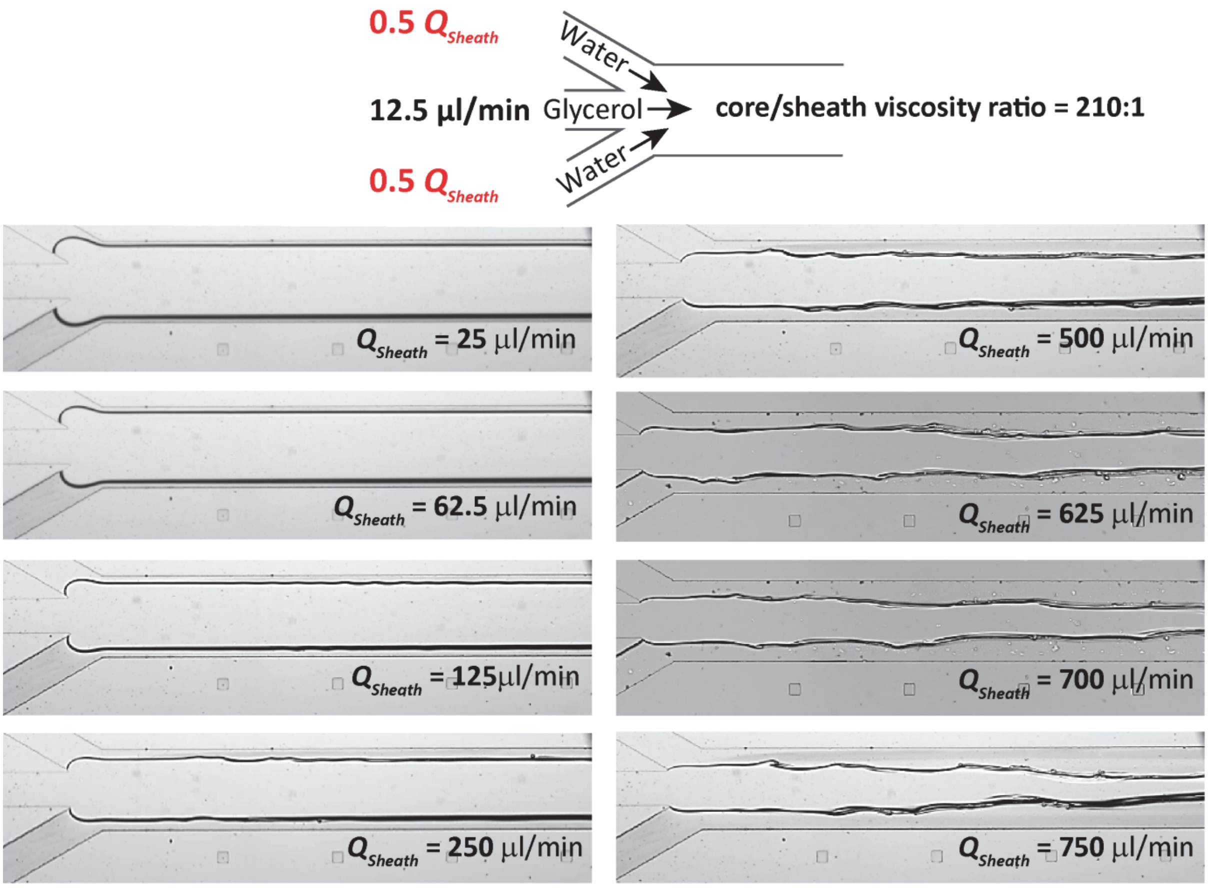

Figure S7a. Dynamics of core-sheath flows under 'stable core' and 'disturbed core' regimes, obtained by setting Q_{core} = 12.5 µl/min while Q_{sheath} = 25 to 750 µl/min.

Supplementary Information 7b: Dynamics of core-sheath flows obtained at μ_{core}/μ_{sheath} = 210, Q_{core} = 12.5 µl/min

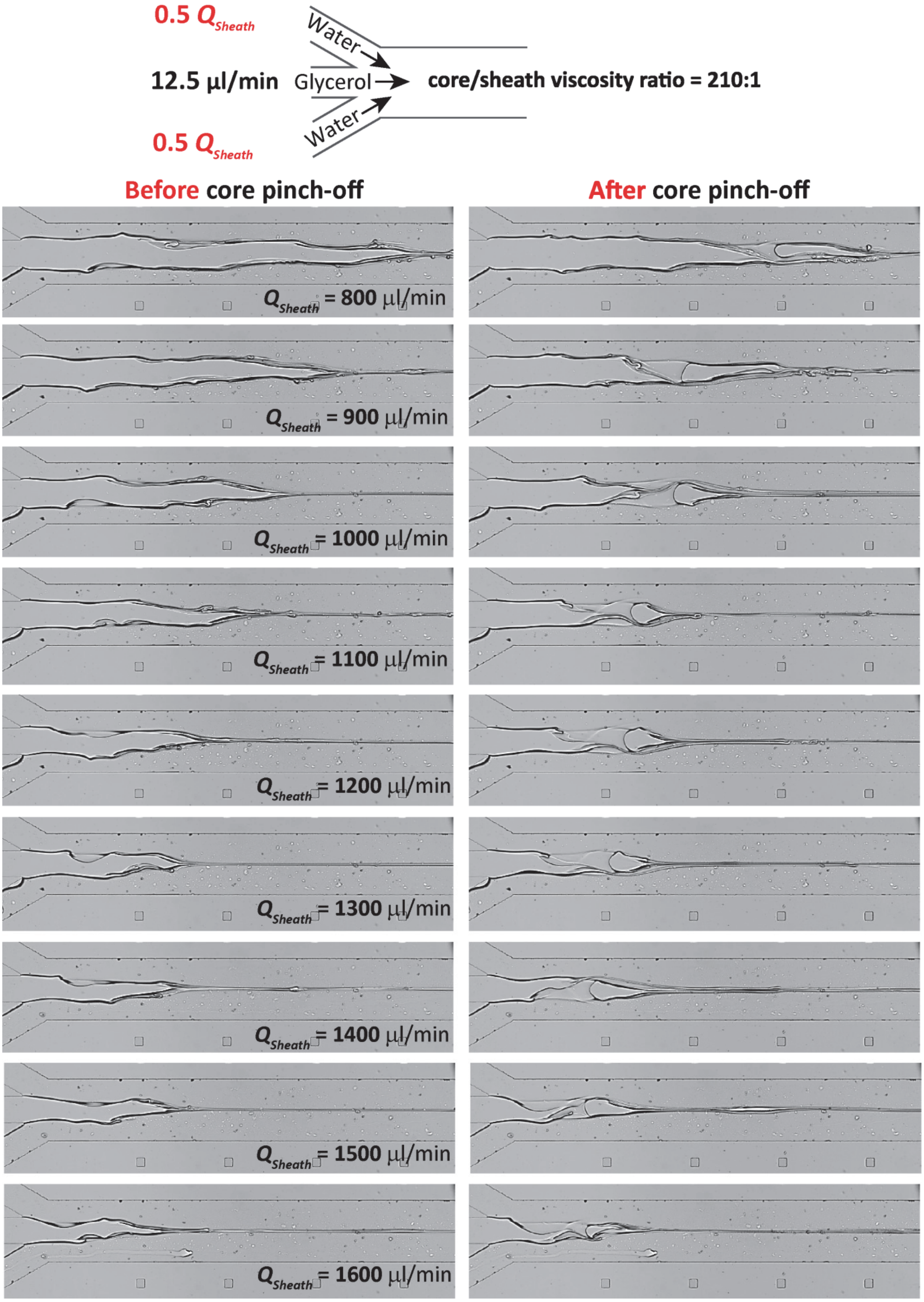

Figure S7b. Dynamics of core-sheath flows under 'broken core' regime, obtained by setting Q_{core} = 12.5 µl/min while Q_{sheath} = 800 to 1600 µl/min.

Supplementary Information 7c: Dynamics of core-sheath flows obtained at μ_{core}/μ_{sheath} = 210, Q_{core} = 12.5 µl/min

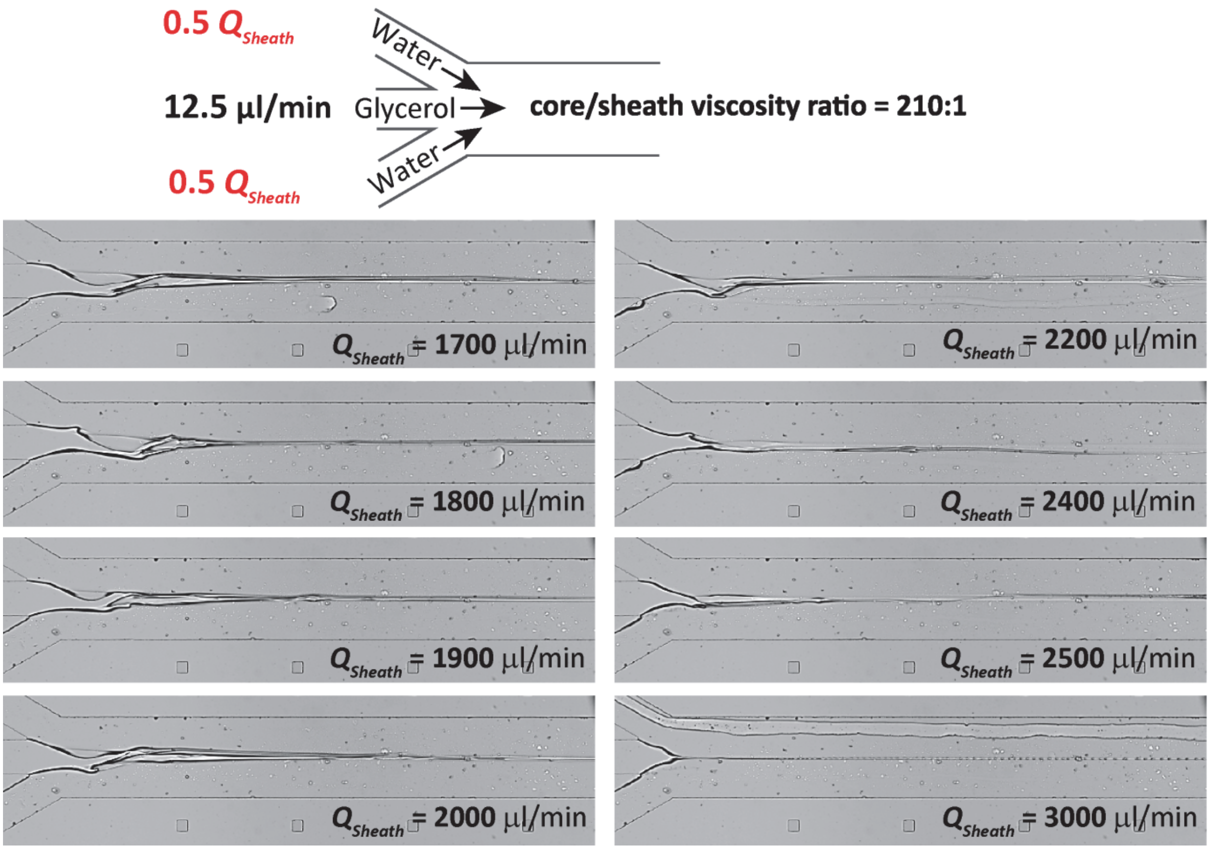

Figure S7c. Dynamics of core-sheath flows under 'oscillating core' regime, obtained by setting Q_{core} = 12.5 µl/min while Q_{sheath} = 1700 to 3000 µl/min.

Supplementary Information 8: Numerical simulation of core/sheath flow structures at μ_{core}/μ_{sheath} = 210, Q_{core} = 12.5 μl/min, Q_{sheath} = 800 μl/min

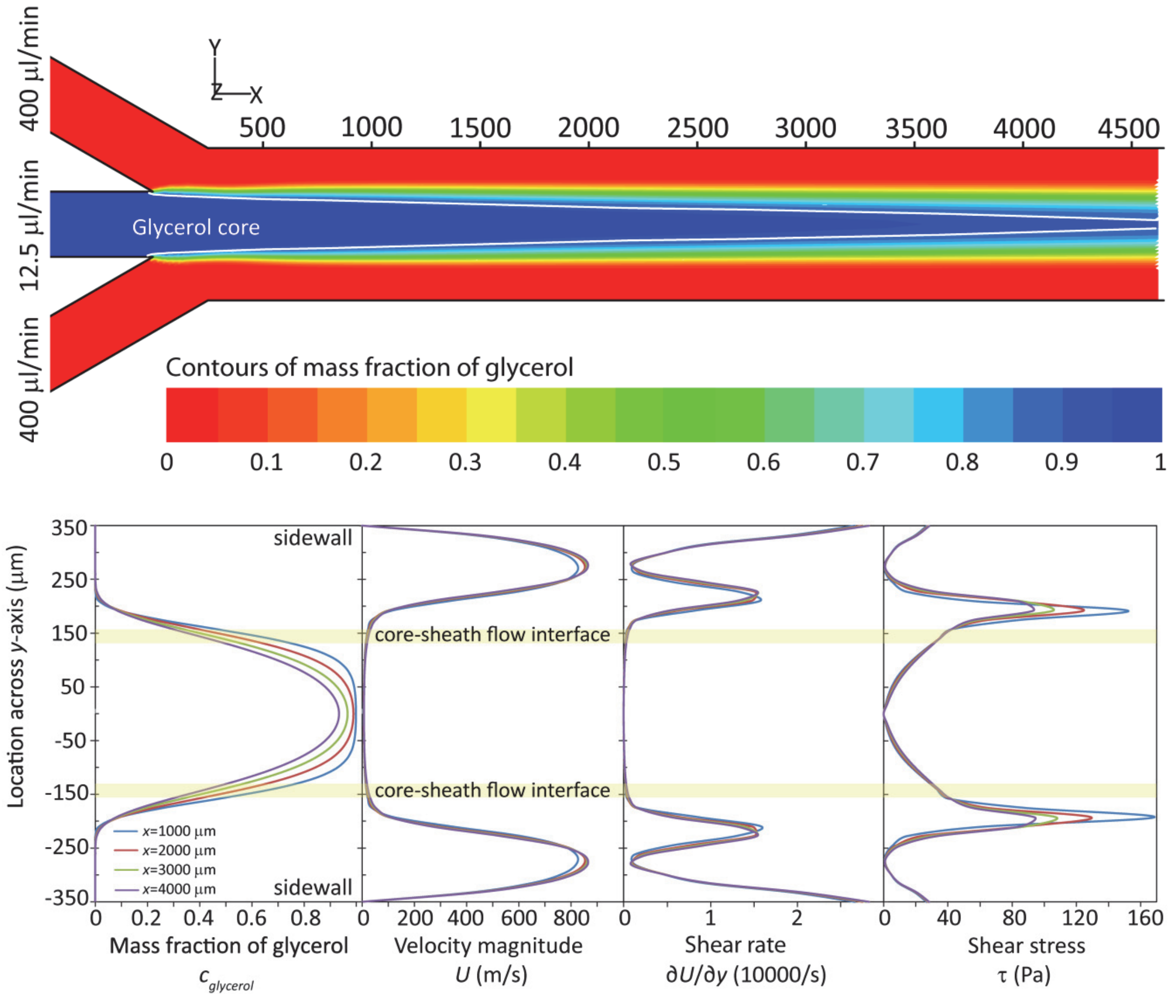

Figure S8. Numerical simulation of core-sheath flows obtained at μ_{core}/μ_{sheath} = 210, Q_{core} = 12.5 μl/min, Q_{sheath} = 800 μl/min (corresponding to 'broken core' regime). Results show the contours of glycerol mass fraction at the bottom surface of the channel, along with variations of glycerol mass fraction, velocity magnitude, velocity gradient (shear rate) and shear stress along the width of the flow focusing channel.

Supplementary Information 9: Dynamics of glycerol-water flows obtained at μ_{core}/μ_{sheath} = 210, $Q_{glycerol}$ = 25 µl/min

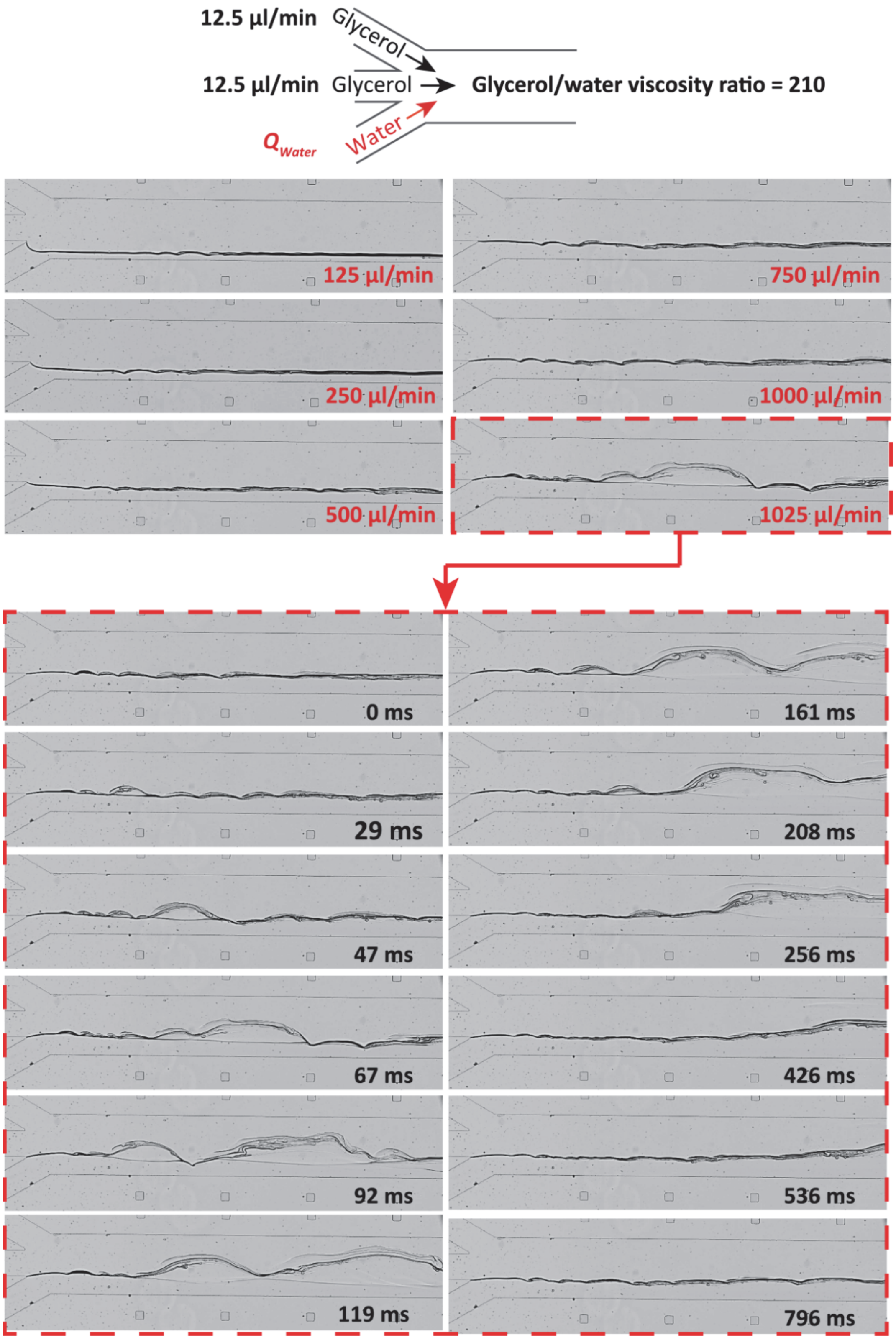

Figure S9. Dynamics of glycerol-water under obtained by setting $Q_{glycerol}$ = 12.5 µl/min through each inlet while varying Q_{water} from 125 to 1025 µl/min, indicating the induction of severe 'interfacial waves' at Q_{water} = 1025 µl/min.

Supplementary Information 10: Dynamics of core-sheath flows obtained at $\mu_{core} / \mu_{sheath}$ = 60, Q_{core} = 1 µl/min

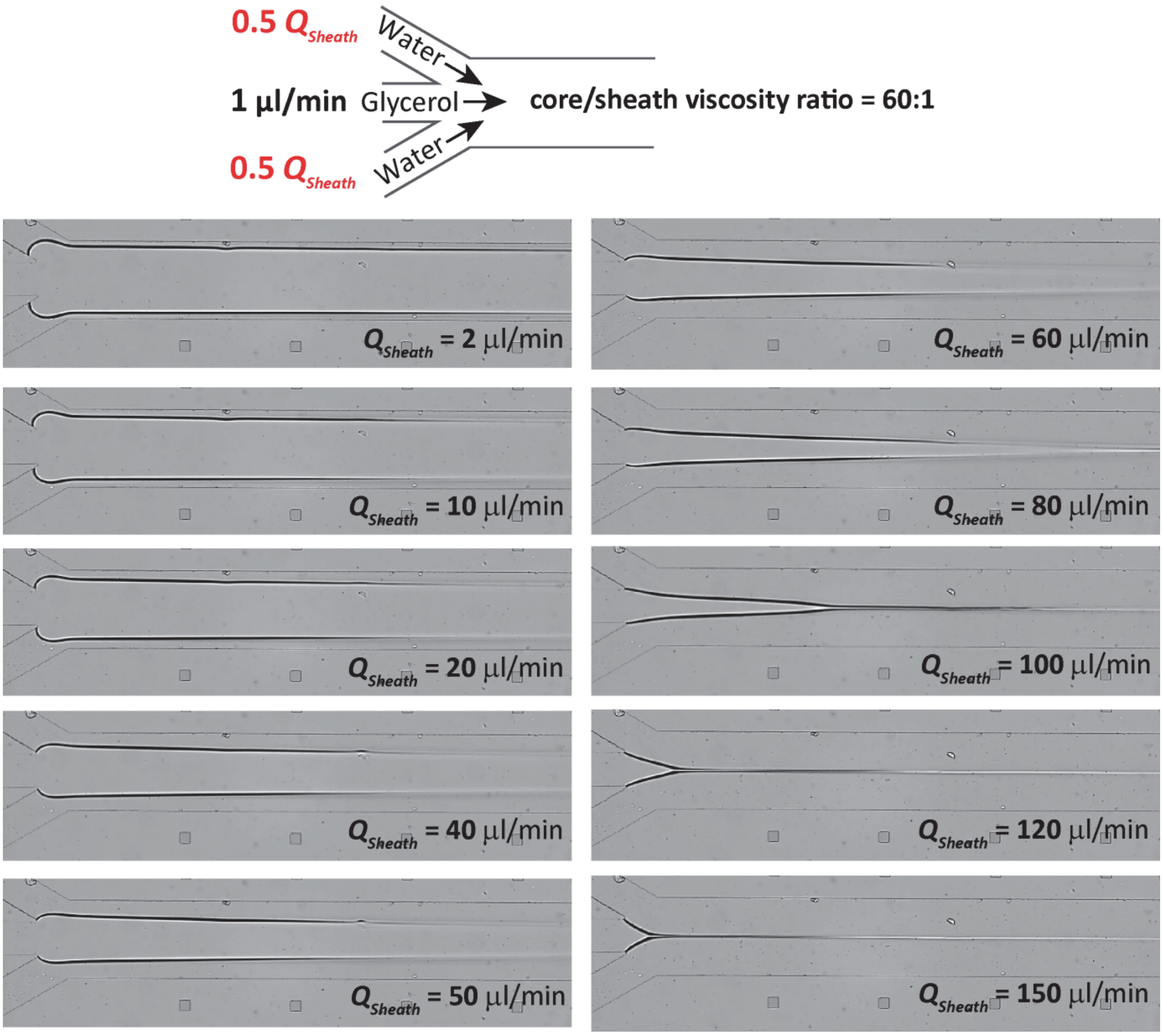

Figure S10. Dynamics of core-sheath flows under 'stable core' regime, obtained by setting Q_{core} = 1 µl/min while Q_{sheath} = 2 to 150 µl/min.

Supplementary Information 11: Dynamics of core-sheath flows obtained at $\mu_{core}/\mu_{sheath} = 60$, $Q_{core} = 5$ µl/min

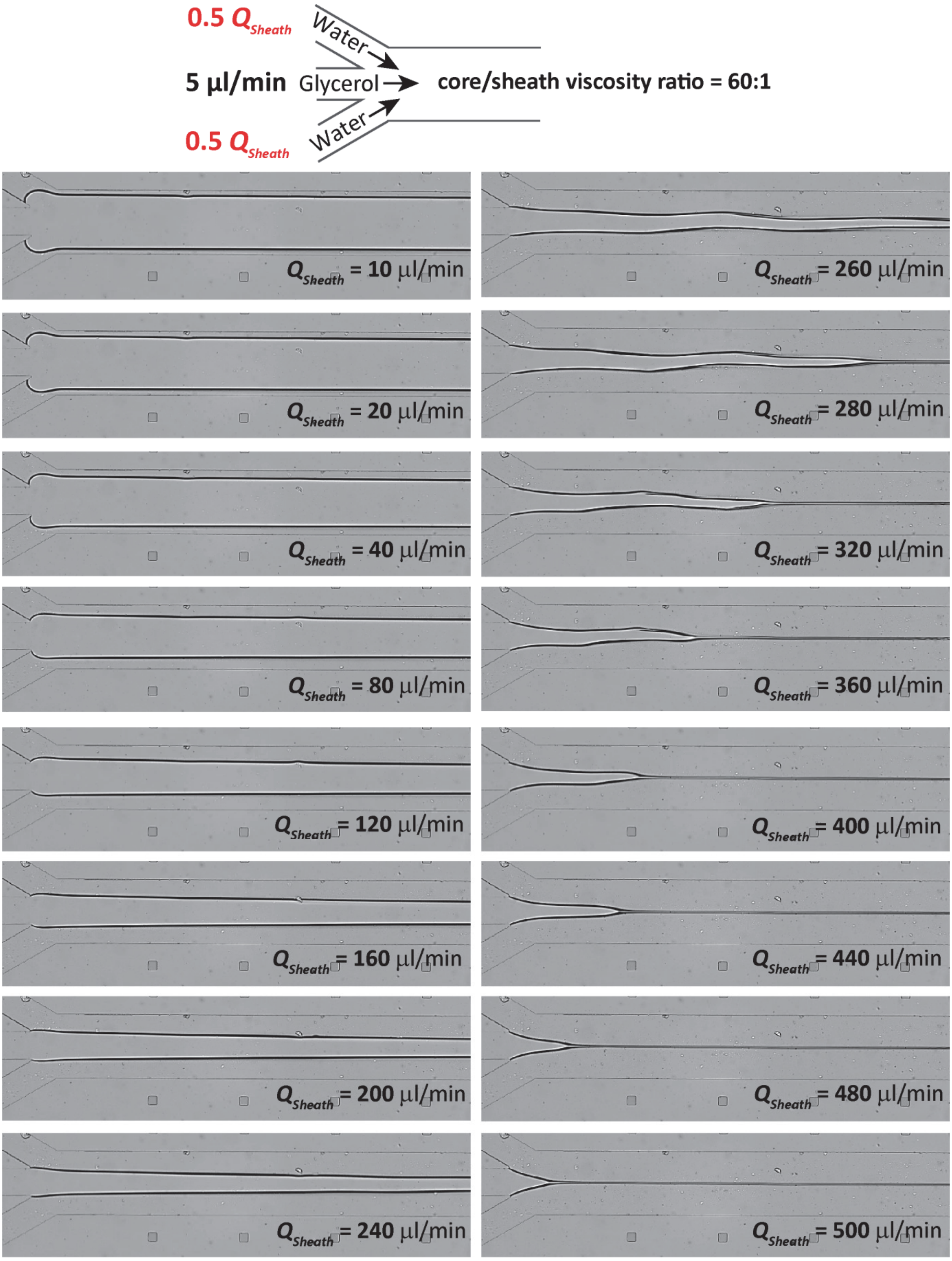

Figure S11. Dynamics of core-sheath flows under 'stable core' and 'disturbed core' regimes, obtained by setting $Q_{core} = 5$ µl/min while $Q_{sheath} = 10$ to 500 µl/min.

Supplementary Information 12: Dynamics of core-sheath flows obtained at $\mu_{core}/\mu_{sheath} = 60$, $Q_{core} = 12.5$ µl/min

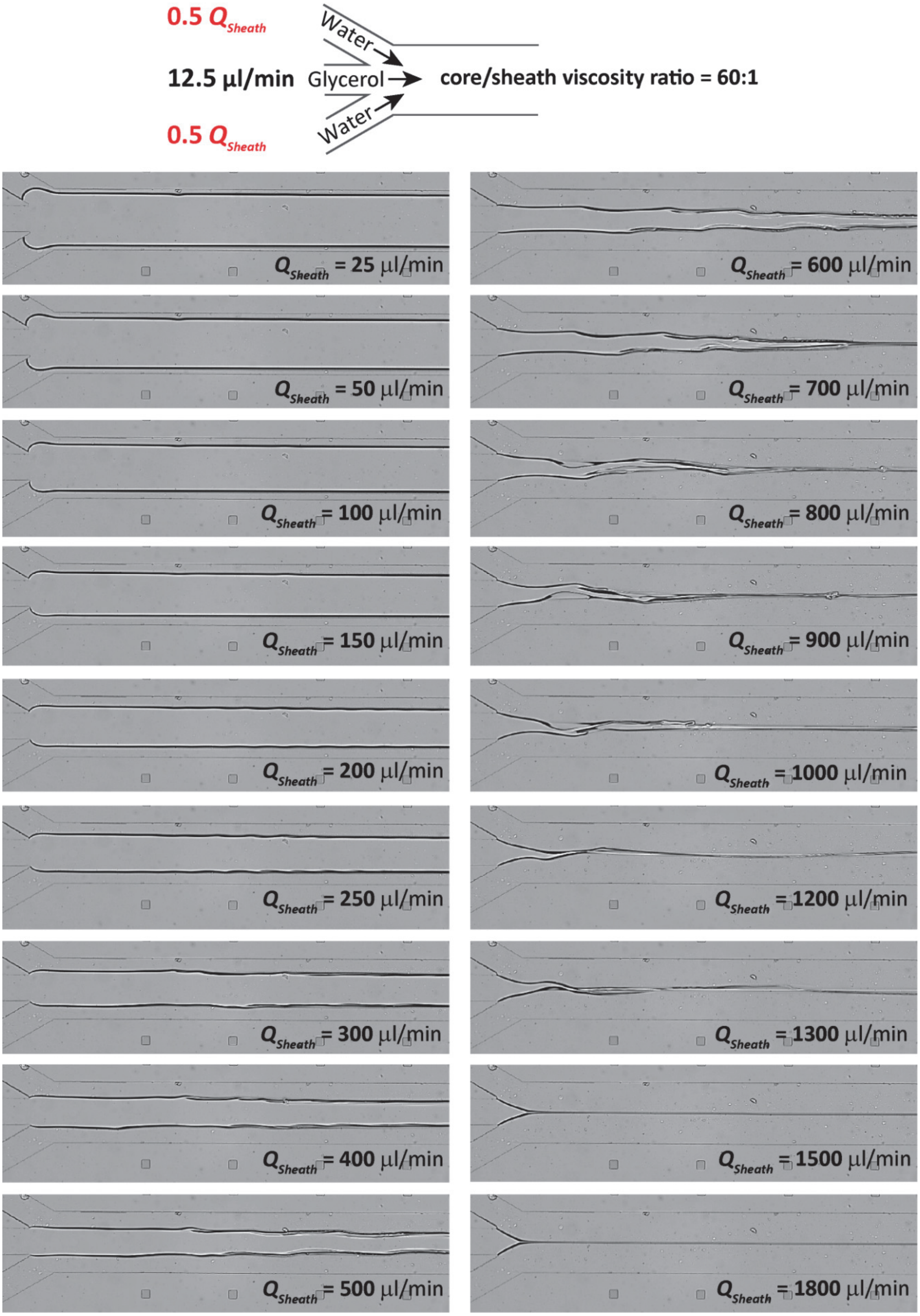

Figure S12. Dynamics of core-sheath flows under 'stable core' and 'disturbed core' regimes, obtained by setting $Q_{core} = 12.5$ µl/min while $Q_{sheath} = 25$ to 1800 µl/min.

Supplementary Information 13a: Dynamics of core-sheath flows obtained at μ_{core}/μ_{sheath} = 60, Q_{core} = 25 μl/min

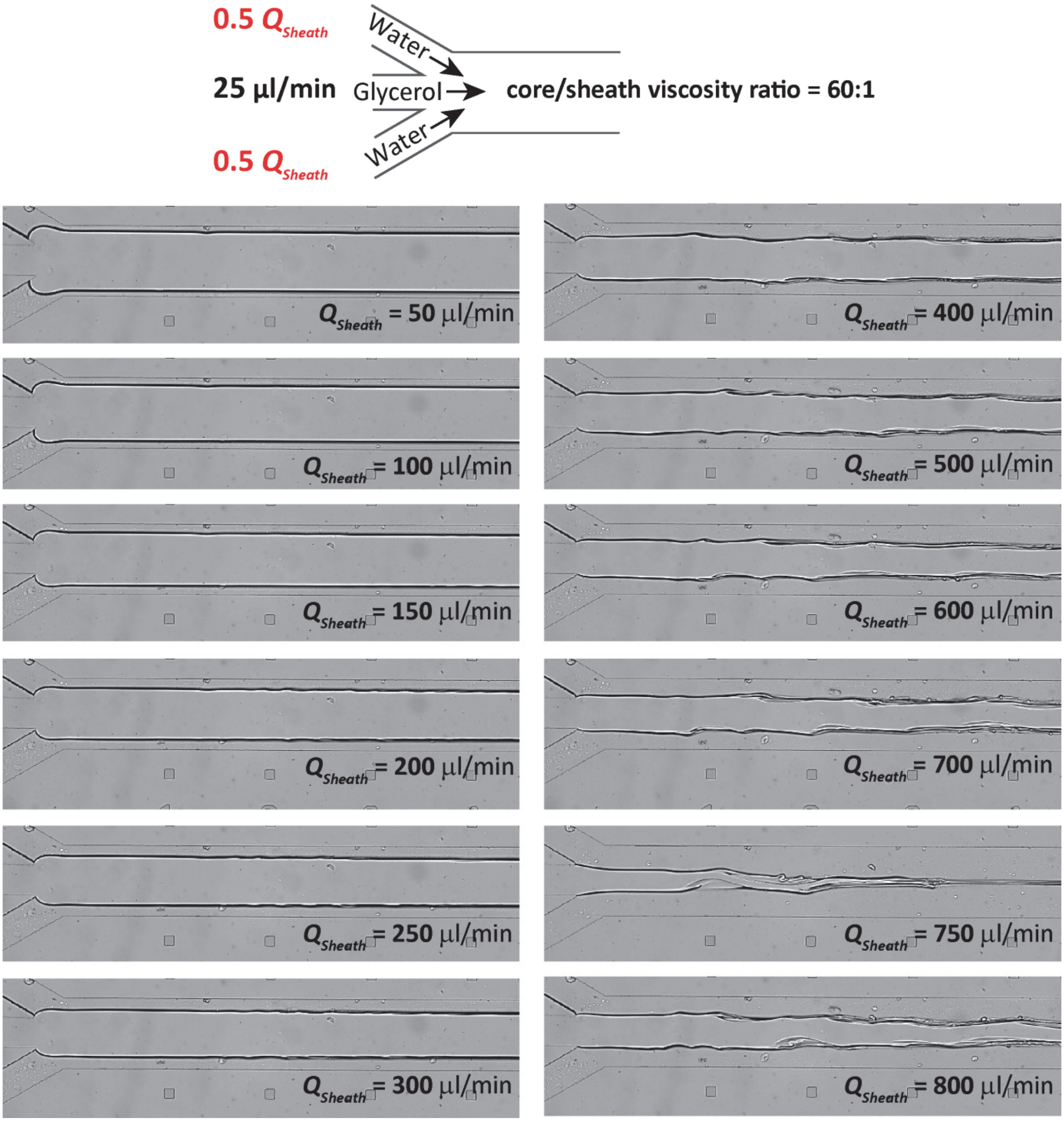

Figure S13a. Dynamics of core-sheath flows under 'stable core' and 'disturbed core' regimes, obtained by setting Q_{core} = 12.5 μl/min while Q_{sheath} = 50 to 800 μl/min.

Supplementary Information 13b: Dynamics of core-sheath flows obtained at μ_{core}/μ_{sheath} = 60, Q_{core} = 25 µl/min

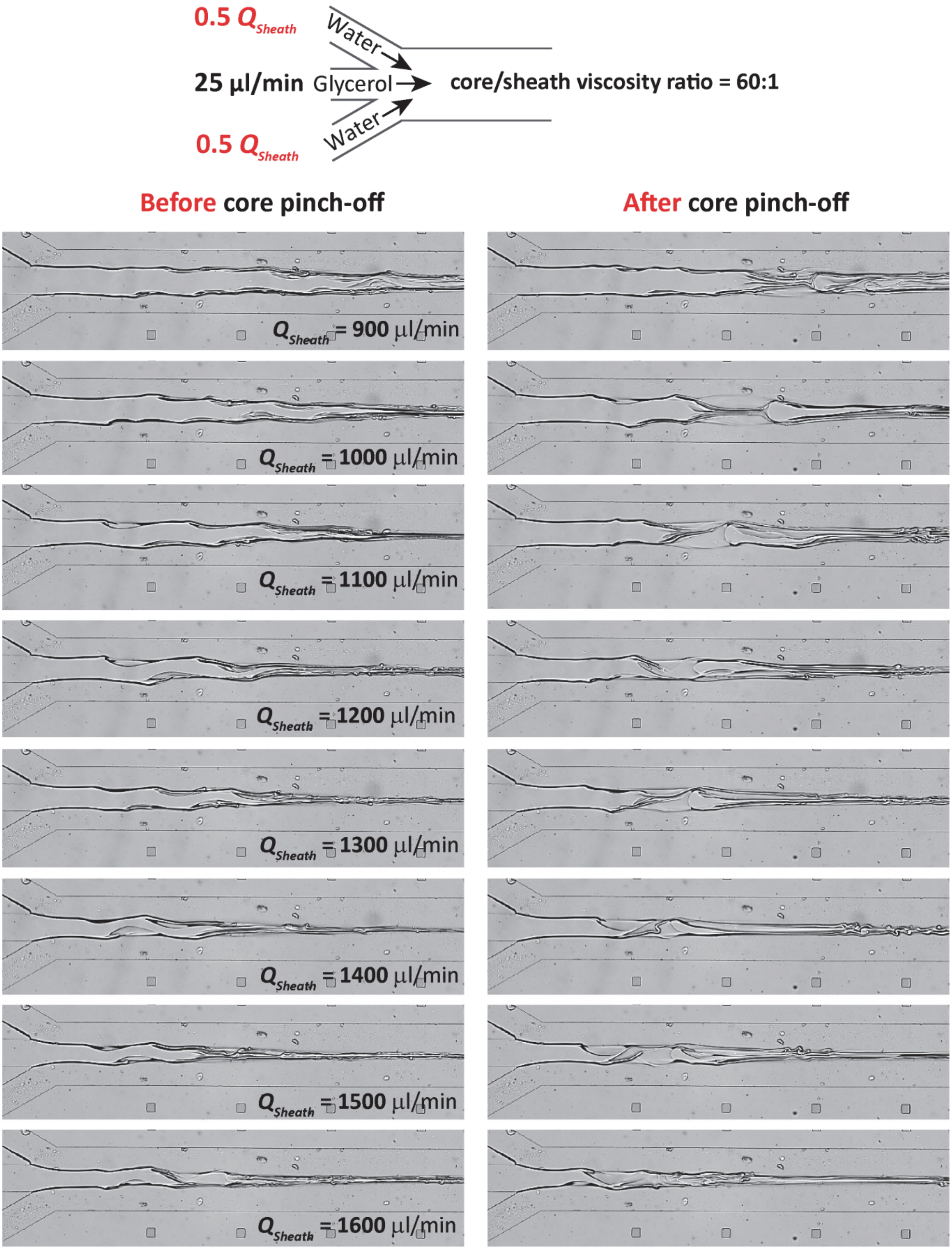

Figure S13b. Dynamics of core-sheath flows under 'broken core' regime, obtained by setting Q_{core} = 12.5 µl/min while Q_{sheath} = 900 to 1600 µl/min.

Supplementary Information 13c: Dynamics of core-sheath flows obtained at μ_{core}/μ_{sheath} = 60, Q_{core} = 25 µl/min

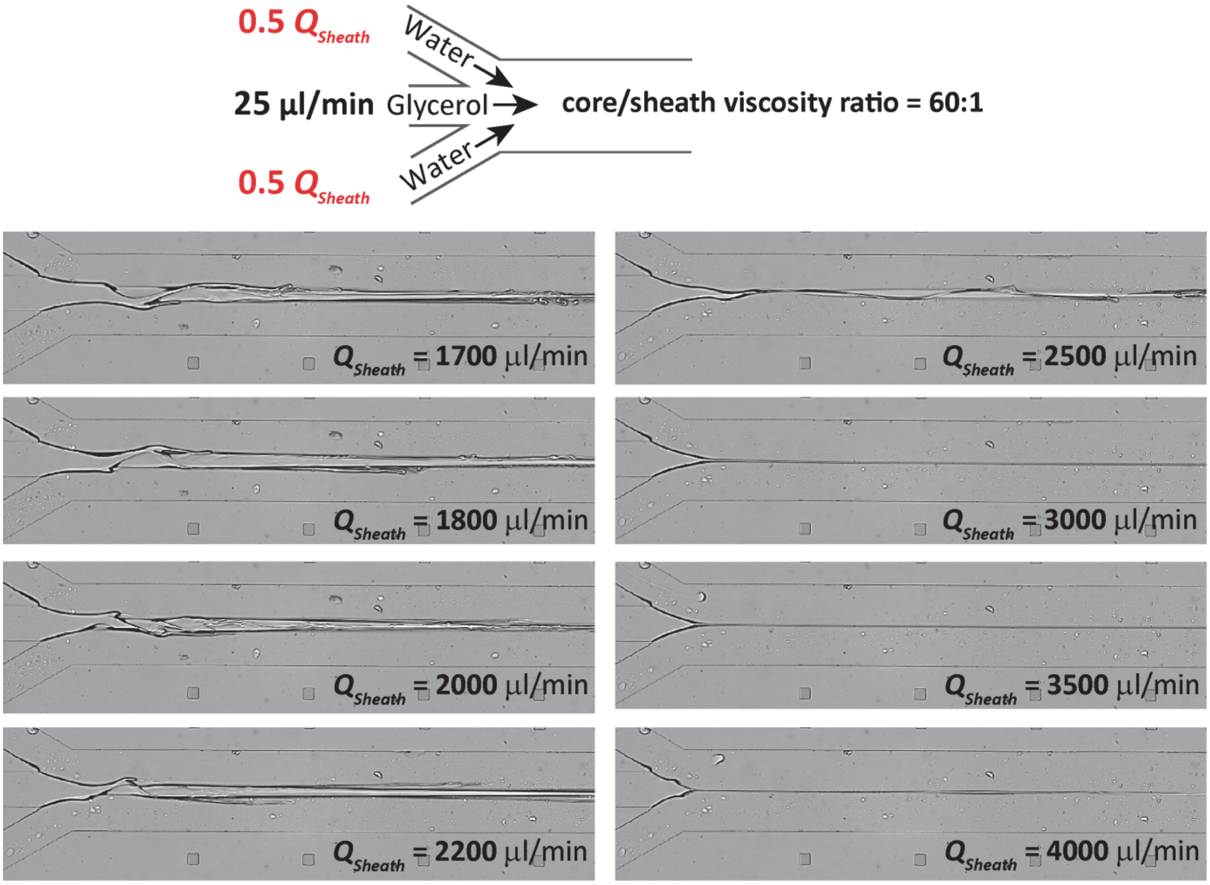

Figure S13c. Dynamics of core-sheath flows under 'oscillating core' regime, obtained by setting Q_{core} = 25 µl/min while Q_{sheath} = 1700 to 4000 µl/min.

Supplementary Information 14: Variations of L_{core}/H and Re $(L_{core}/H)^4$ against Re for various core-sheath flow combinations obtained at $\mu_{core}/\mu_{sheath} = 60$

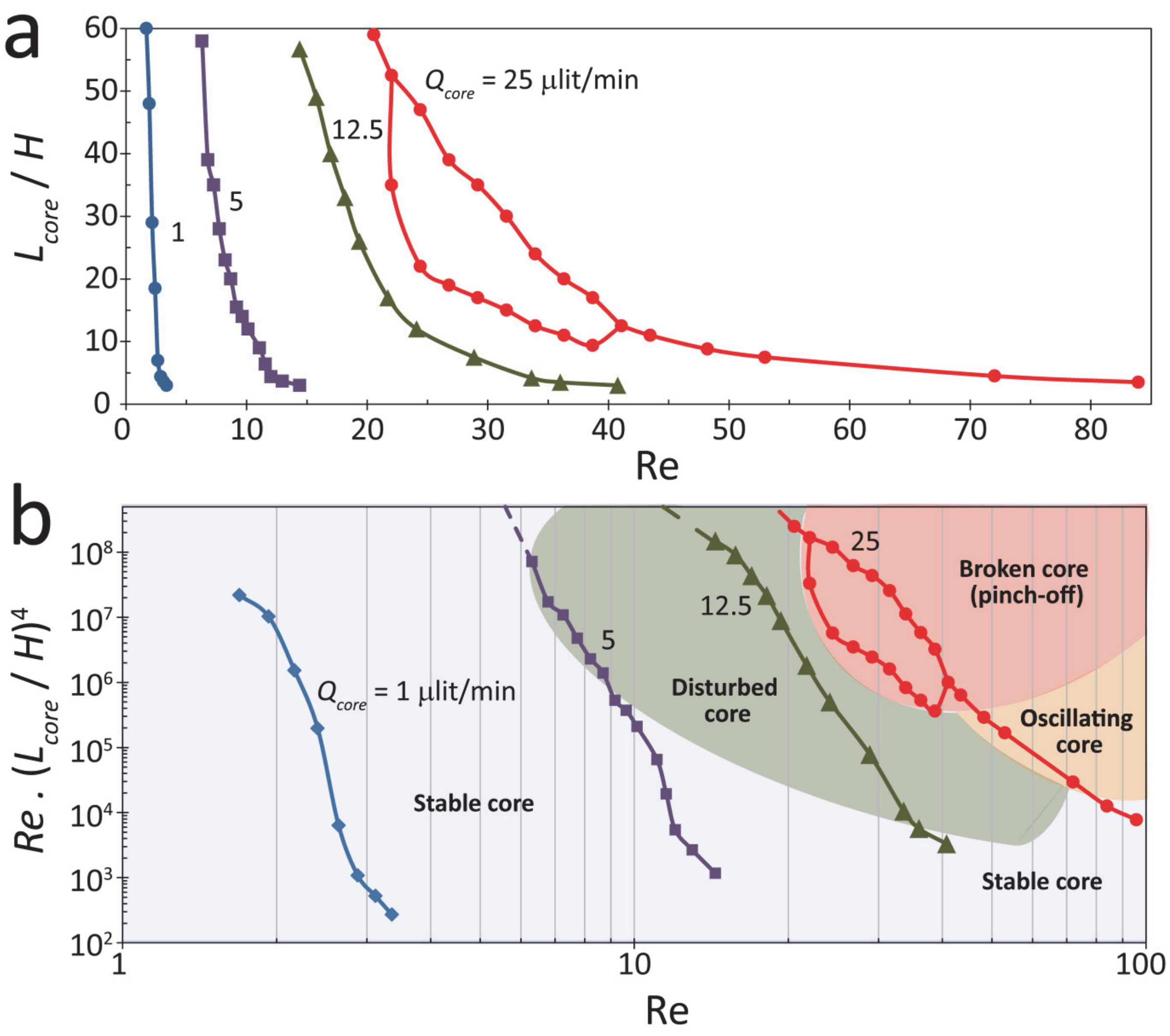

Figure S14: Variations of L_{core}/H and Re $(L_{core}/H)^4$ against Re for various core-sheath flow combinations obtained at $\mu_{core}/\mu_{sheath} = 60$. Similar to the case of $\mu_{core}/\mu_{sheath} = 210$, the dynamics of highly viscous core flow can be classified into 'stable core', 'disturbed core', 'broken core' and 'oscillating core' regimes.
